# NUP155 insufficiency recalibrates a pluripotent transcriptome with network remodeling of a cardiogenic signaling module

**DOI:** 10.1186/s12918-018-0590-x

**Published:** 2018-05-30

**Authors:** Claudia C. Preston, Saranya P. Wyles, Santiago Reyes, Emily C. Storm, Bruce W. Eckloff, Randolph S. Faustino

**Affiliations:** 1 Genetics and Genomics Group, Sanford Research, 2301 E. 60th Street N, Sioux Falls, SD 57104 USA; 2 Department of Dermatology, Mayo Clinic, 200 1st St SW, Rochester, MN 55905 USA; 3 Department of Surgery, Wake Forest University Health Sciences, Medical Center Boulevard, Winston-Salem, NC 27157 USA; 4 Medical Genome Facility, Mayo Clinic, 200 1st St SW, Rochester, MN 55905 USA; 5 Department of Pediatrics, Sanford School of Medicine of the University of South Dakota, 1400 W. 22nd Street, Sioux Falls, SD 57105 USA

**Keywords:** NUP155, Atrial fibrillation, RNAseq, Embryonic stem cells, Network bioinformatics

## Abstract

**Background:**

Atrial fibrillation is a cardiac disease driven by numerous idiopathic etiologies. NUP155 is a nuclear pore complex protein that has been identified as a clinical driver of atrial fibrillation, yet the precise mechanism is unknown. The present study employs a systems biology algorithm to identify effects of NUP155 disruption on cardiogenicity in a model of stem cell-derived differentiation.

**Methods:**

Embryonic stem (ES) cell lines (*n* = 5) with truncated NUP155 were cultured in parallel with wild type (WT) ES cells (*n* = 5), and then harvested for RNAseq. Samples were run on an Illumina HiSeq 2000. Reads were analyzed using Strand NGS, Cytoscape, DAVID and Ingenuity Pathways Analysis to deconvolute the NUP155-disrupted transcriptome. Network topological analysis identified key features that controlled framework architecture and functional enrichment.

**Results:**

In NUP155 truncated ES cells, significant expression changes were detected in 326 genes compared to WT. These genes segregated into clusters that enriched for specific gene ontologies. Deconvolution of the collective framework into discrete sub-networks identified a module with the highest score that enriched for Cardiovascular System Development, and revealed NTRK1/TRKA and SRSF2/SC35 as critical hubs within this cardiogenic module.

**Conclusions:**

The strategy of pluripotent transcriptome deconvolution used in the current study identified a novel association of NUP155 with potential drivers of arrhythmogenic AF. Here, NUP155 regulates cardioplasticity of a sub-network embedded within a larger framework of genome integrity, and exemplifies how transcriptome cardiogenicity in an embryonic stem cell genome is recalibrated by nucleoporin dysfunction.

**Electronic supplementary material:**

The online version of this article (10.1186/s12918-018-0590-x) contains supplementary material, which is available to authorized users.

## Background

Electrical disorders in the heart are a hallmark feature of a class of clinical cardiac pathologies called arrhythmias that are the underlying substrate for heart failure, stroke and sudden cardiac death [1, 2]. The most common sustained arrhythmia observed in a clinical setting is atrial fibrillation (AF), with a population prevalence that increases with aging [3]. AF is defined as a sustained supraventricular tachyarrhythmia with disorganized atrial activation and ineffective contraction that has distinctive electrocardiogram characteristics including: fast atrial rate of ~ 300 beats/min; absence of P waves; and irregular R-R intervals [1, 4]. This type of sustained arrhythmia is accompanied by co-morbidities in the elderly, where the majority of this cohort presents with concomitant structural alterations of the heart [3]. Even though AF is more prevalent in octogenarians, a percentage of patients less than 60 years of age appear in the clinic with a “healthy heart” history. These individuals are diagnosed with idiopathic or lone AF, an unexplained arrhythmia where clinical studies report inconclusive or negative results [5]. Studies to address this gap in knowledge have focused on ion channel gene variants, however recent work has attributed significant contributions of several non-ion channel substrates to AF [6–8]. Among these, nucleoporins (nups) have emerged as potential epigenomic regulatory proteins.

Nups comprise the nuclear pore complexes (NPCs), which are large toroidal structures with a main function of directing the selective transport of macromolecules between the cytoplasm and the nucleus. NPCs were first described in *Xenopus* oocytes using electron microscopy, with modern understanding of its intricate structure and morphology revealed by advanced techniques such as cryo-electron microscopy (cryo-EM) and super-resolution microscopy [9, 10]. NPCs are composed of cytoplasmic, inner, and nucleoplasmic rings, each of which consists of multiple copies of nups stacked and linked together to form distinct NPC subcomplexes [10, 11]. Furthermore, interactions among discrete NPC subcomplexes create specialized structural and functional domains within the pore. For example, the Y-subcomplex is a well characterized component of the NPC that interacts with the inner ring subcomplex to form the NPC scaffold [12]. Recent studies in eukaryotes have revealed that apart from their canonical function as architectural components of the pore and nucleocytoplasmic transport mediators, nups play a significant role in regulation of transcriptional activity and chromatin structure/organization that impacts phenotype [13–15]. Indeed, nup-driven differentiation is conserved among a variety of metazoans, where nups play active roles in development [16–18].

Moreover, altered nup dynamics have been associated with normal and pathologic cardiogenesis, with a range of clinical phenotypes that range from morphological defects to contractile and electrical impairment of heart function [19, 20]. For example, some components of the NPC inner ring subcomplex affect nuclear localization and histone acetylation of the *HOXA* gene cluster that underlies mesodermal development and proper cardiac morphology [21, 22]. Disrupted NUP188 results in congenital heart defects (CHD) associated with left-right patterning disorders [23]. Idiopathic and dilated cardiomyopathy, in which myocardial function is progressively impaired, is associated with NDC1, NUP160, NUP153, NUP93, and NUP62 expression changes that together disrupt nuclear transport [20, 24]. Intracellular acidification associated with ischemic cardiac disease was reported to be regulated in part by NUP35 through its ability to bind the 5’ UTR of *nhe1*, an mRNA that encodes a sodium-hydrogen exchanger essential for maintaining pH homeostasis in cardiomyocytes [25]. NUP155, being a critical scaffolding component of the NPC, in a homozygous mutant form has been shown to impair atrial electrical signaling and give rise to clinical atrial fibrillation [19]. This presents as ectopic initiation of contraction, reentrant impulses, and futile cycling that ultimately compromises cardiac function and leads to sudden death in early childhood [19]. In contrast to the nup-associated cardiopathologies described above, the precise cellular and molecular mechanisms by which NUP155 contributes to supraventricular arrhythmias such as atrial fibrillation remains unknown.

Systems and network biology algorithms can identify cryptogenic drivers of AF through high throughput dataset cartography. This approach has been used to profile cardiogenic transcriptome changes and capture remodeled intermolecular relationships to identify categories of functional perturbation in a cardiopathological model of differentiation [26, 27]. Here, a network-based bioinformatic strategy was applied to decipher complex systems biology impacts of NUP155 disruption in an embryonic stem cell line that models mammalian arrhythmogenesis. This work is the first to characterize pluripotent transcriptome remodeling regulated by NUP155, where we identify novel and high value NUP155-regulated candidates associated with AF etiology. Significantly, transcriptome networks that arise from NUP155 insufficiency revealed alterations in membrane function and extracellular interactions, and specifically identified NTRK1/TRKA and SRSF2/SC35 as hubs essential to the integrity of a cardiogenic sub-network.

## Methods

All media and reagents have been procured from Fisher Scientific, unless specifically noted. NUP155-deficient embryonic stem cell lines along with wild type parental cell lines were obtained from Bay Genomics (Berkeley, CA).

### Embryoid body formation and Ca^2+^ imaging

Embryoid body (EB) formation and imaging to measure Ca^2+^ transients in contractile EBs was performed as previously described [26, 28]. Mouse embryonic stem (ES) cells were maintained in Glasgow’s Minimum Essential Medium (GMEM, Gibco) supplemented with penicillin G/streptomycin (Pen/Strep), sodium pyruvate (Lonza BioWhittaker), non-essential amino acids (NEAA, Corning), β-mercaptoethanol (β-ME, Sigma-Aldrich), 7.5% fetal bovine serum (FBS, EMD Millipore) and ESGRO leukemia inhibitory factor (LIF, EMD Millipore), and passaged three times to establish stable growth before embryoid body (EB) formation. ES cell lines were differentiated into three-layered EBs using the hanging-drop method. Briefly, cells were harvested and resuspended in differentiation medium that contained 20% FBS without LIF, to a concentration of 8 × 10^4^ cells/ml. To facilitate EB formation, hanging drops were created by depositing 25 μl of the cell suspension on the lids of 500-cm2 square culture plates and incubated for 48 h. To induce spontaneous differentiation, EBs were flushed and transferred to floating suspension for another 48 h. Following differentiation, cells were cultured in differentiation media containing GMEM supplemented with Pen/Strep, sodium pyruvate, NEAA, β-ME and 20% FBS. Alternatively, Aggrewell (STEMCELL Technologies Inc., Cambridge, MA) plates were used to promote uniform EB size and organization and were cultured in differentiation media as described above. EBs were grown for three days, with media changes as necessary before transferring to gelatin coated dishes. Beating foci could be observed between 5 and 7 days after plating.

Ca^2+^ imaging: Contractile EBs were incubated in Tyrode’s solution at 37 °C and then loaded with the Ca^2+^ indicator dye, Fluo-4-AM (5 μM) for 15 min. Stained EBs were imaged with a Zeiss LSM Live 5 laser confocal microscope (Zeiss, Oberkochen, Germany). Spontaneous Ca^2+^ transients were recorded at 37 °C using ZEN 2.1 software (Zeiss, Oberkochen, Germany), and plotted as a function of time using Excel (Microsoft, Redmond, WA).

### Cell culture and RNA extraction

Wild type (WT) and NUP155 exon truncated E14TG2a.4 (NUP155^+/−^) feeder independent mouse ES cell lines were cultured on 0.1% gelatin coated 100 mm dishes grown in 10 ml of GTES medium consisting of 85% Glasgow MEM (GMEM), 15% ES qualified Fetal Bovine Serum (FBS), sodium pyruvate, non-essential amino acids (NEAA), penicillin/streptomycin (PenStrep), β-mercaptoethanol (β-ME) and ESGRO Leukemia Inhibitory Factor (LIF). After initial plating (seeding density at 1.0 × 10^6^–1.5 × 10^6^ cells), cells were maintained in culture for 2–3 passages, changing GTES media as required. At approximately 80% confluency, cells were passaged by treatment with 5 ml of 0.25% trypsin for 4 min at 37 °C. Trypsin digestion was arrested by addition of an equal amount of GTES media. This suspension was centrifuged at 1500 rpm for 4 min, and pellets of cells were either resuspended in GTES media to be re-plated or in PBS prior to RNA extraction. Total RNA was extracted with an RNeasy Mini kit according to manufacturer’s protocol (Qiagen, Germantown, MD) in preparation for sequencing on HiSeq 2000 System (Illumina, San Diego, CA).

### Transcriptome deconvolution

Interrogation of previously published Gene Expression Omnibus (GEO) dataset ID# GDS3729 was performed via Genespring GX to prioritize cardiogenic nup candidates. To determine *nup* genes that demonstrated consistent and significant changes in expression during cardiogenesis, differential gene expression analysis and self-organizing map (SOM) classification were independently performed between undifferentiated LIF+ ES cells (ES LIF+) and stem cell-derived cardiomyocytes (CM). For increased resolution of WT and NUP155-deficient ES transcriptomes, five independent biological replicates of WT and NUP155^+/−^ lines (*n* = 10 total) were submitted for RNAseq. RNA libraries were prepared according to the manufacturer’s instructions for the TruSeq RNA Sample Prep Kit v2 (Illumina, San Diego, CA) from 100 ng of total RNA. Briefly, polyA mRNA was purified from total RNA using oligo dT magnetic beads. Purified mRNA was fragmented at 95 °C for 8 min and eluted from the beads. Double stranded cDNA was prepared using SuperScript III reverse transcriptase, random primers (Invitrogen, Thermo Fisher Scientific, Waltham, MA) and DNA polymerase I and RNase H. The cDNA ends were repaired and an “A” base added to the 3′ ends. TruSeq paired end index DNA adaptors (Illumina, San Diego, CA) with a single “T” base overhang at the 3′ end were ligated and resulting constructs were purified using AMPure SPRI beads (Agencourt Bioscience, Beverly, MA). The adapter-modified DNA fragments were enriched by 12 cycles of PCR using Illumina TruSeq PCR primers (Illumina, San Diego, CA). The concentration and size distribution of the libraries were determined using an Agilent Bioanalyzer DNA 1000 chip (Agilent Technologies, Santa Clara, CA) and Qubit fluorometry (Invitrogen, Thermo Fisher Scientific, Waltham, MA).

Libraries were sequenced at 5 samples per lane to generate 70–90 million reads per sample following Illumina’s standard protocol using the Illumina cBot and cBot Paired end cluster kit version 3. The flow cells were sequenced as 101 × 2 paired end reads on an Illumina HiSeq 2000 using TruSeq SBS sequencing kit version 3 and HCS v2.0.12 data collection software. Base-calling was performed using Illumina’s RTA v1.17.21.3. This data was deposited into the NIH GEO database with accession number GSE111596.

### Determination of loss of function intolerance metrics and differential expression analysis

The Exome Aggregation Consortium (ExAC) browser was used to investigate pathological potential of our identified nups [29]. Data extracted from the ExAC browser included probability of loss of function (LoF) intolerance (pLI) metric, and z scores for missense metrics [30].

For RNAseq bioinformatics, raw reads (as *.bam files) were imported into Strand NGS for expression analysis (Agilent Technologies, Santa Clara, CA). Samples were aligned to the *Mus musculus* genome (Build mm10) and annotated using RefSeq (Release 80). Unmatched paired-end reads were filtered out for downstream quality control. These datasets were further refined by removing reads that did not reach a mapping quality above 20, as well as those that did not surpass vendor-established quality control (QC) criteria in Strand NGS (Agilent Technologies, Santa Clara, CA).

Final QC reads were normalized by DESeq with median of all samples used as baseline. All reads represented a total of 36,172 gene entities that were indexed according to fold change and statistical significance, using the criteria of a 2.0-fold change (or greater) and possessing a *p*-value of 0.05 or less to identify a quality filtered transcriptome, for a total of 326 genes.

### Gene ontology analysis, and network cartography

This signature transcriptome was separated into upregulated (176 entities) and downregulated (150 entities) groups for functional annotation, KEGG, and Reactome pathway enrichment analysis using DAVID Bioinformatics Resources [31–34]. To determine over representation or enrichment, the DAVID algorithm employs a modified Fisher’s exact test that is incorporated into a score that reports relative priority [32]. Gene lists defined for each cluster were submitted to DAVID using Entrez Gene identifiers for downstream analyses. The highest classification stringency was selected to maintain robust groups and scores were reported for KEGG and Reactome pathways when applicable.

To map functional interactions among genes within the quality filtered transcriptome, the total gene list was submitted to Ingenuity Pathway Analysis (IPA; Qiagen, Germantown, MD) to identify subnetworks within the transcriptome, and construct an integrated de novo gene regulatory network (GRN) to determine overall functional priorities and network topology. A total of 10 subnetworks were identified that were assembled into one inclusive network using the “Merge Networks” function within IPA. Edges within this collective network indicate functional interactions among genes, supported by published empirical observations curated within the IPA database. These relationship data were collated and exported in .xls format using the “Export Data ➔ Export ➔ All Relationships” feature within IPA, and served as an input file for network analysis in Cytoscape [35]. Graph theory metrics, to quantify network structure and topology, were determined using the “Network Analyzer” tool in Cytoscape, and data was used to prioritize gene targets for further analysis.

## Results

### Discrete nucleoporin gene expression changes in cardiogenesis

**Fig. 1:**
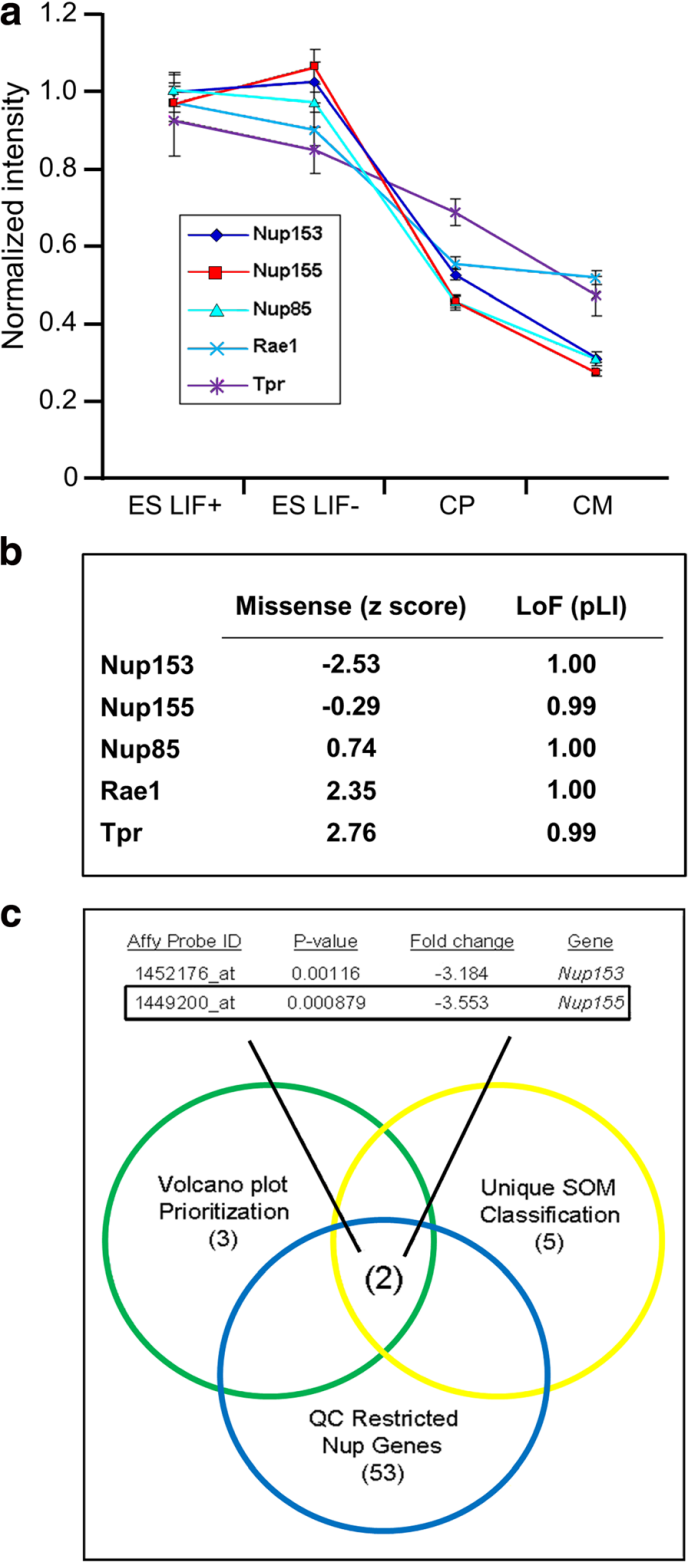
Nucleoporins in cardiac differentiation. **a** *Nup153, Nup155, Nup85, Rae1,* and *Tpr* expression profiles during cardiac specification. Undifferentiated stem cells (ES LIF+); early differentiated stem cells (ES LIF-); cardiac precursors (CP), cardiomyocytes (CM). **b** ExAC constraint metrics and pLI values for *Nup153, Nup155, Nup85, Rae1,* and *Tpr*. **c** Venn diagrams intersect discrete gene groups independently parsed from the same high throughput dataset to identify nucleoporins (nup) that emerge as robust candidates involved in cardiogenesis. *Nup155* is the highest priority molecule (*p* < 0.001). SOM = self-organizing map; QC = quality control

Cardiac fate is regulated by temporospatial gene expression [26], and nucleoporins are emerging as key players in the determination of cardiac structure and function. Previous gene expression analysis in a model of stem cell-derived cardiogenesis revealed global down-regulation of nuclear transport genes with cardiac differentiation [36]. To gain novel insights into nup expression dynamics in cardiogenesis, we performed a nup-focused SOM cluster analysis of our original GEO dataset GDS3729 and identified a unique nup gene set that contained *Nup153, Nup155, Nup85, Rae1,* and *Tpr.* (Fig. 1a). Analysis of these genes using the.

Exome Aggregation Consortium (ExAC) browser revealed that only *Nup155* and *Nup153* possessed negative missense constraint Z-scores (more variants than expected) with probability of loss of function (LoF) intolerance (pLI) metrics of > 0.9, which infers extreme LoF intolerant genes, while the remaining three NPC proteins (*Nup85, Rae1,* and *Tpr*) had a positive Z-score (inferred as increased constraints with fewer variants) with high pLI (Fig. 1b). Furthermore, *Nup155* and *Nup153* demonstrated consistent significance confirmed by independent volcano plot and quality control thresholding analyses. Of these, *Nup155* emerges as the most significantly changed nucleoporin transcript (*p* = 0.000879), downregulated by more than 3.5-fold in stem cell-derived cardiomyocytes (Fig. 1c).

### Dysrhythmia of NUP155 deficient contractile embryoid bodies

**Fig. 2:**
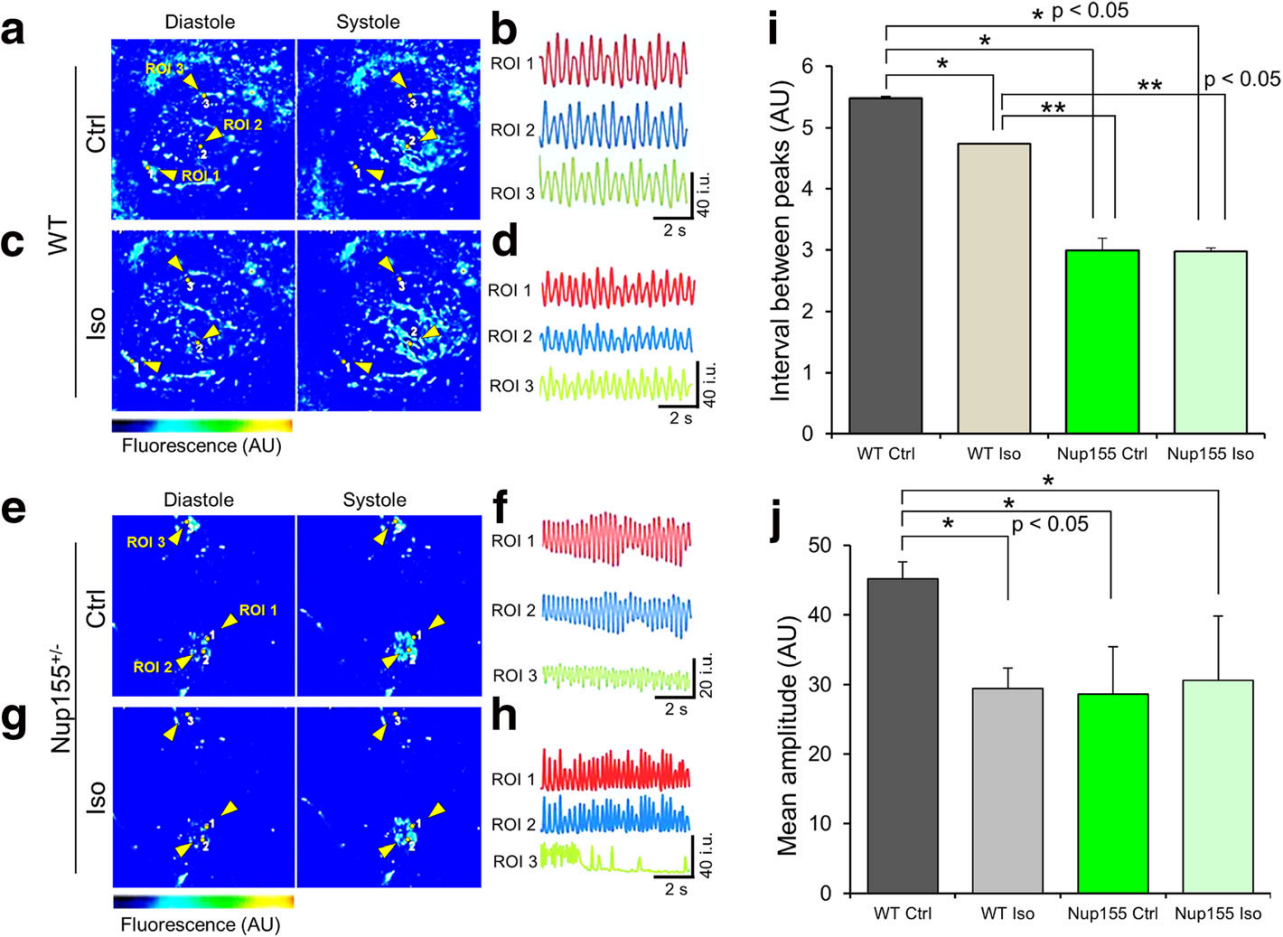
Contractile NUP155 deficient embryoid bodies exhibit dysrhythmia. **a** Embryoid bodies (EBs) were loaded with Fluo4-AM to visualize Ca^2+^ handling during systolic and diastolic phases of contractile cycling, as indicated. Color legend to left of image identifies fluorescence of regions that range from high Ca^2+^ concentration in red, medium concentration in cyan, to low concentration in blue. **b** Ca^2+^ handling for each region of interest (ROI), with timescale in seconds (s) and range of fluorescent intensity units (i.u.) indicated in lower right. **c**, **d** Visualization and measurement of contractile EBs following treatment with 10 mM isoproterenol (Iso). **e**, **f** Untreated beating areas in NUP155 deficient EBs demonstrates increased frequency of contractile cycling, with variable Ca^2+^ handling. **g**, **h** Agonist treatment of NUP155 deficient EBs aggravates the irregular contractile cycling observed in unstimulated controls that ranged in severity from hypercontractility to loss of rhythmic contraction. **I** Measurement of intervals between peaks highlight a significant decrease of time between Ca^2+^ waves in NUP155^+/−^ EBs compared to controls, independent of isoproterenol treatment (*n* = 3, *p < 0.05 vs WT Ctrl, **p < 0.05 vs WT Iso). **j** Changes in mean amplitude of Ca^2+^ waves. WT Iso treated, and NUP155^+/−^ EBs with and without Iso treatment did not show significant differences, but were significantly decreased compared to WT Ctrl. (*n =* 3, *p < 0.05 vs WT Ctrl)

Differentiation of ES cells into beating embryoid bodies (EBs) recapitulates cardiac phenotypes of automaticity and electromechanical coupling (Fig. 2). Fluorescent quantitation of Ca^2+^ transients in wild type control (WT Ctrl) EBs demonstrated constant and regular rhythm (Fig. 2a, b). Treatment of WT Ctrl with the β-adrenergic receptor agonist isoproterenol (Iso, 10 μM) increased the frequency of Ca^2+^ cycling (Fig. 2c, d) depicted by a significant decrease of time between Ca^2+^ signal peaks in WT treated EBs compared with WT Ctrl (Fig. 2i). In contrast, unstimulated control NUP155 deficient (NUP155^+/−^ Ctrl) contractile EBs (Fig. 2e) exhibited drastically increased beating frequency, reminiscent of myocardial fibrillation, with variable and diminished amplitude of Ca^2+^ waves compared to WT EBs (Fig. 2f, j). Isoproterenol stimulation in the NUP155^+/−^ EBs (Fig. 2g) exacerbated the irregularity of the Ca^2+^ waves, with responses that ranged from a blunted chronotropic effect to loss of agonist response (Fig. 2h), however no difference in interval times was observed compared with NUP155^+/−^ Ctrl (Fig. 2i). Taken together these results show that NUP155-deficient EBs are prone to electrical instability which may serve as a substrate for atrial fibrillation.

### Transcriptome remodeling in NUP155-disrupted embryonic stem cells

**Fig. 3:**
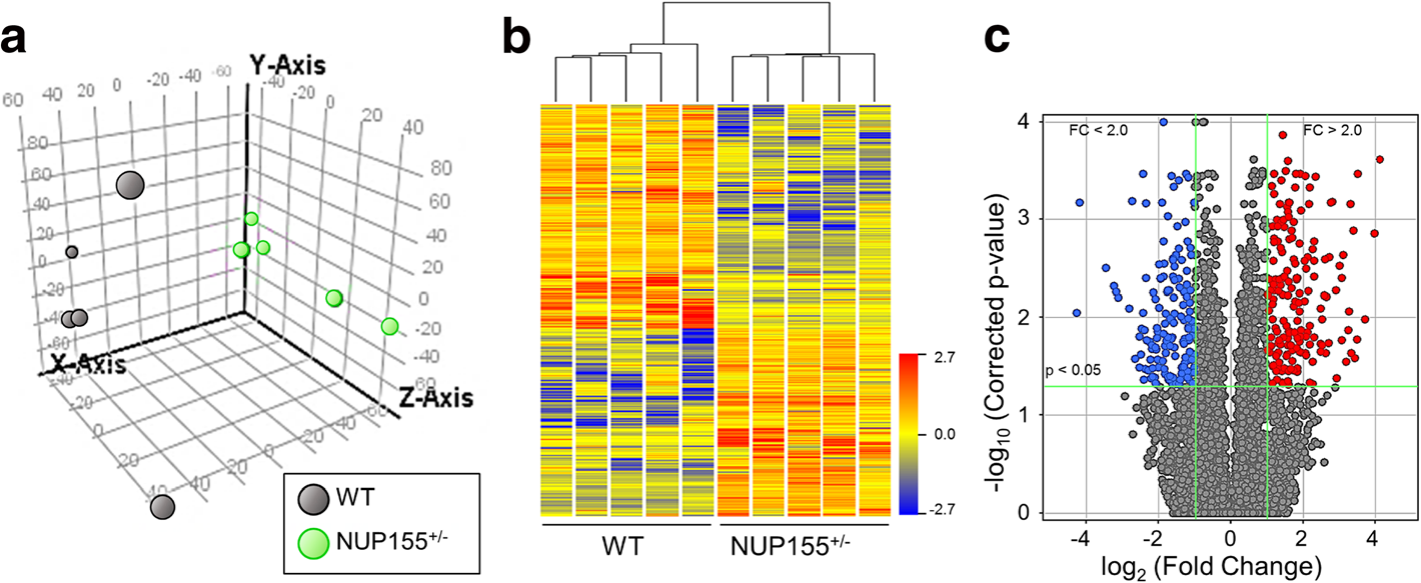
Molecular signature of NUP155 truncation in a pluripotent genome. Deep transcriptome profiling of WT and NUP155^+/−^ embryonic stem cells was performed using RNAseq. **a** Principal component analysis (PCA) revealed distinct hallmark gene expression profiles with clear segregation of WT from NUP155^+/−^ transcriptomes. Filled circles represent ES (dark grey) and NUP155^+/−^ (light green) transcriptomes of distinct biological replicates, plotted in a three dimensional volumetric space. *Axes*: X – PC1 (24.08%), Y – PC2 (13.2%), Z – PC3 (10.38%). **b** Pairwise correlation of samples reveals reproducible clustering of discrete up and downregulated gene expression patterns that define ES and NUP155^+/−^ populations. Lower right: colorscale indicates normalized intensity, where red, yellow, and blue represent upregulated, no change, and downregulated trends, respectively. **c** Volcano plot of gene expression changes to enumerate up and downregulated mRNA in the NUP155^+/−^ transcriptome, according to criteria of absolute Fold Change (FC) > 2.0 and *p* < 0.05. Filled circles in red represent up-regulated genes that meet this criteria, while blue circles represent downregulated transcripts. Circles in grey indicate genes that fall below the indicated threshold values. A total of 326 genes were identified that met the filtering parameters, with 176 up and 150 downregulated, respectively. PC – Principal Component

Dysfunctional contractility in beating EBs is supported by the clinical role of NUP155 in arrhythmogenesis [19], together with previous reports that have identified gene activation and repression associated with NUP155 in neonatal rat ventricular myocytes [37]. These data suggest a broad capacity for NUP155 to remodel global gene expression profiles in a cardiac setting. To investigate the effects of NUP155 in a cardiogenic context, mouse ES cells that harbor disrupted NUP155 were examined by RNAseq to understand transcriptome changes precipitated by NUP155 in a pluripotent background. Principal component analysis (PCA) plots distinguished WT from NUP155^+/−^ transcriptomes (Fig. 3a). Hierarchical clustering of individual transcriptomes demonstrated clear segregation and reproducibility of gene expression profiles for each biological sample category (Fig. 3b). Replicate analysis was performed to delimit transcripts to those changing by 2.0 fold or greater as well as meeting significance criteria of *p* < 0.05. A total of 176 and 150 up and downregulated genes were identified that met these criteria (Fig. 3c). Pathway enrichment analysis using DAVID [31] identified several thematic clusters for up (14 clusters) and downregulated (11 clusters) gene lists (Additional file 1: Tables S1 and S2). The significant functional terms, depicted in Table 1 (*p* ≤ 0.05), included functions related to integrin alpha for both up and downregulated genes (Upregulated and Downregulated Clusters 1), protein phosphorylation (Upregulated Cluster 2), transmembrane tyrosine protein kinase activity (Up Cluster 3), and cysteine switch/zinc binding (Upregulated Cluster 7, Table 1).

**Table 1:** Pathway enrichment analysis of differentially expressed genes

	Database	Term	Description	*p*-Value	FE
Upregulated					
Cluster 1 (1.84)	Uniprot Seq Feature	short sequence motif	GFFKR motif	0.005	27.897
	InterPro	IPR018184	Integrin alpha chain, C-terminal cytoplasmic region, conserved site	0.007	22.714
		IPR000413	Integrin alpha chain	0.008	21.452
		IPR013649	Integrin alpha-2	0.008	21.452
		IPR013517	FG-GAP repeat	0.008	21.452
		IPR013519	Integrin alpha beta-propeller	0.009	20.323
	SMART	SM00191	Integrin alpha	0.012	17.511
	EMBL-EBI	GO:0008305	Integrin complex	0.016	15.321
Cluster 2 (1.50)	InterPro	IPR000719	Protein kinase, catalytic domain	0.019	2.499
		IPR011009:	Protein kinase-like domain	0.029	2.315
	EMBL-EBI	GO:0004672	Protein kinase activity	0.029	2.314
		GO:0006468	protein phosphorylation	0.040	2.180
		GO:0016310	phosphorylation	0.054	2.052
Cluster 3 (1.35)	InterPro	IPR020635	Tyrosine-protein kinase, catalytic domain	0.025	6.356
		IPR008266	Tyrosine-protein kinase, active site	0.041	5.201
	SMART	SM00219	TyrKc, Tyrosine kinase, catalytic domain	0.036	5.477
	EMBL-EBI	GO:0007169	transmembrane receptor protein tyrosine kinase signaling pathway	0.045	5.023
	UniProtKB		Tyrosine-protein kinase	0.052	4.750
Cluster 7 (0.64)	Uniprot Seq Feature	metal ion-binding site	Zinc binding site, in inhibited form	0.024	12.505
		short sequence motif	Cysteine switch	0.041	9.299
Downregulated					
Cluster 1 (1.84)	Uniprot Seq Feature	short sequence motif	GFFKR motif	0.005	27.897
	InterPro	IPR018184	Integrin alpha chain, C-terminal cytoplasmic region, conserved site	0.007	22.714
		IPR000413	Integrin alpha chain	0.008	21.452

Pathway enrichment analysis of significantly up and downregulated genes in Nup155^+/−^ ES cells performed with DAVID. Here are depicted the clusters with functional terms that reached significance of p ≤ 0.05. Each cluster shows their respective enrichment score in parentheses. Database represents the online functional database used to extract each term; Term represents the pathway identification; Description is the pathway symbol; and FE refers to the fold enrichment score

### Molecular cartography prioritizes genes within transcriptome subnetworks

**Fig. 4:**
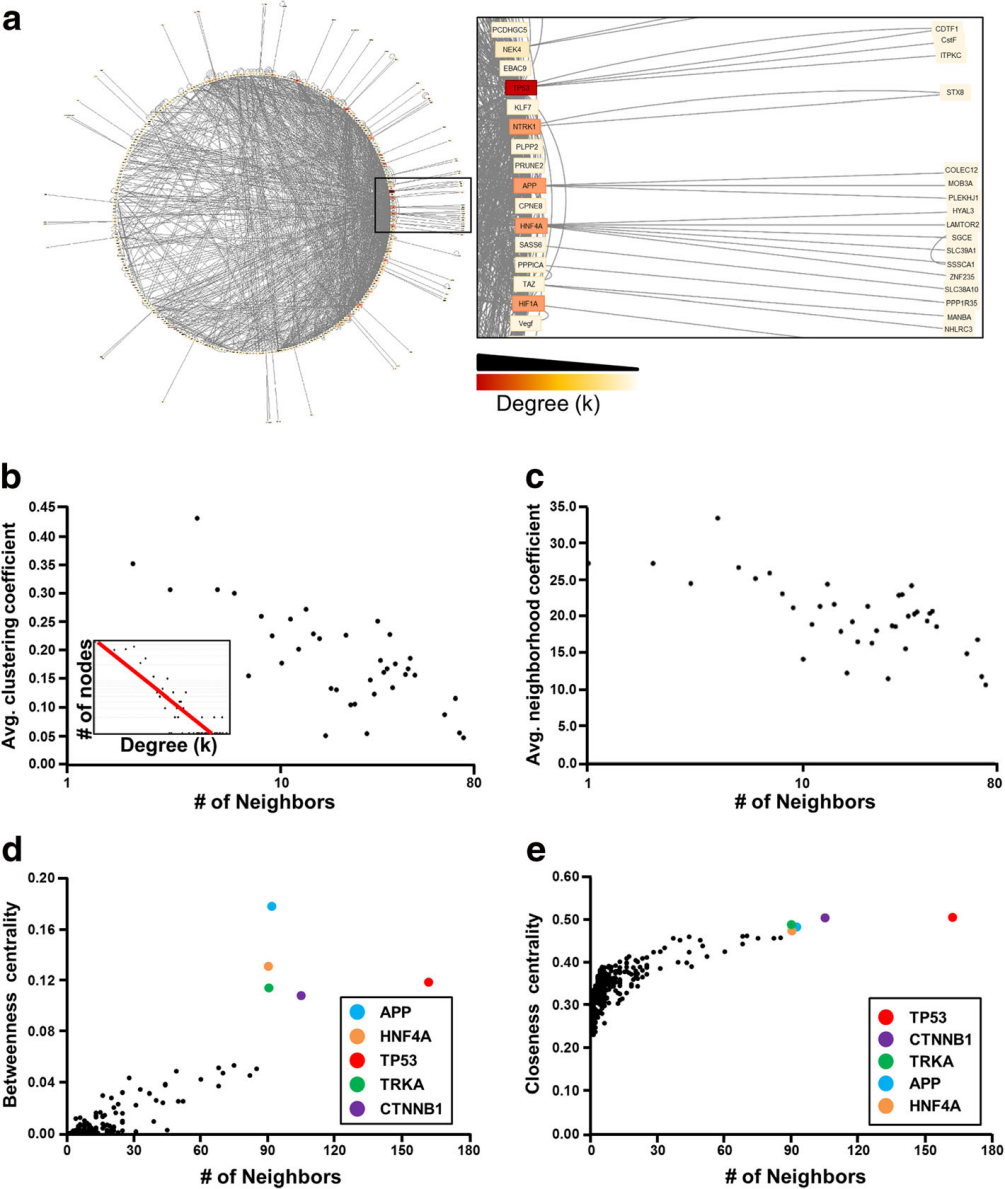
Network cartography of a nucleoporin-disrupted transcriptome. (**a**) The collective transcriptome inclusive of up and downregulated transcripts were analyzed by Ingenuity Pathways Analysis to identify experimentally observed interactions among the 326 genes. The network generated from this data was visualized in a circular layout that positions nodes circumferentially with their connections (edges) plotted diametrically. Singletons are network nodes with only one connection to the larger network and are arrayed on the outside of the circle plot. This layout emphasizes nodes that have high edge density, seen in this network on the right. *Right panel*: Magnification of network arc with high edge density. Nodes were colored properties according to degree, or number of connections, where high degree is represented by dark red and low degree in white, shown here in the colorscale above panel. **b** Topological analysis revealed a clustering coefficient distribution associated with hierarchical network structure. *Inset:* Degree distribution demonstrates a power law relationship indicative of scale-free architecture (**c**) Neighborhood connectivity plot identifies dissortative nature of the network, where highly connected nodes tend to connect to nodes with a lower number of edges. **d** Nodes with high betweenness centrality are critical to maintaining network integrity as they connect other regions of the network to one another. APP, HNF4A, TP53, NTRK1/TRKA, and CTNNB1 possessed distinct betweenness centrality scores that segregated them from other nodes in the network. *Inset:* Legend identifies genes with the topmost betweenness centrality scores, ranked in order from highest to lowest, and are colored to facilitate identification within the plot. **e** Closeness centrality scores are important for speed of informational transmission within a network. Here, nodes with the highest closeness centrality clustered together. The nodes prioritized for high betweenness centrality measures were identical to the molecules with critical closeness centrality scores. *Inset:* Legend depicts nodes rank ordered from high to low. Identity of nodes with discrete centrality metrics are preserved as identical, yet distinct reprioritization of those molecules is observed on comparison of closeness versus betweenness

Up and downregulated genes were integrated using Ingenuity Pathway Analysis to form a collective de novo gene regulatory network. Gene relationships were extracted and exported for analysis in Cytoscape where they were visualized in a circular layout to emphasize nodes with high edge density (Fig. 4a). Topology analysis confirmed scale-free and hierarchical properties innate to biological networks (Fig. 4b and ***inset***). Neighborhood connectivity (Fig. 4c), betweenness centrality (Fig. 4d) and closeness centrality (Fig. 4e) plots of the integrated NUP155 transcriptome identified preferential attachment nature and bridging nodes key to network connectivity and information flow. The highest betweenness scores in this NUP155-remodeled transcriptome were, in order of priority: APP, HNF4A, TP53, NTRK1, and CTNNB1 (Fig. 4d), whereas the top closeness centrality scores were associated with the same 5 molecules in a different order of priority, i.e. TP53, CTNNB1, NTRK1, APP, and HNF4A (Fig. 4e).

**Fig. 5:**
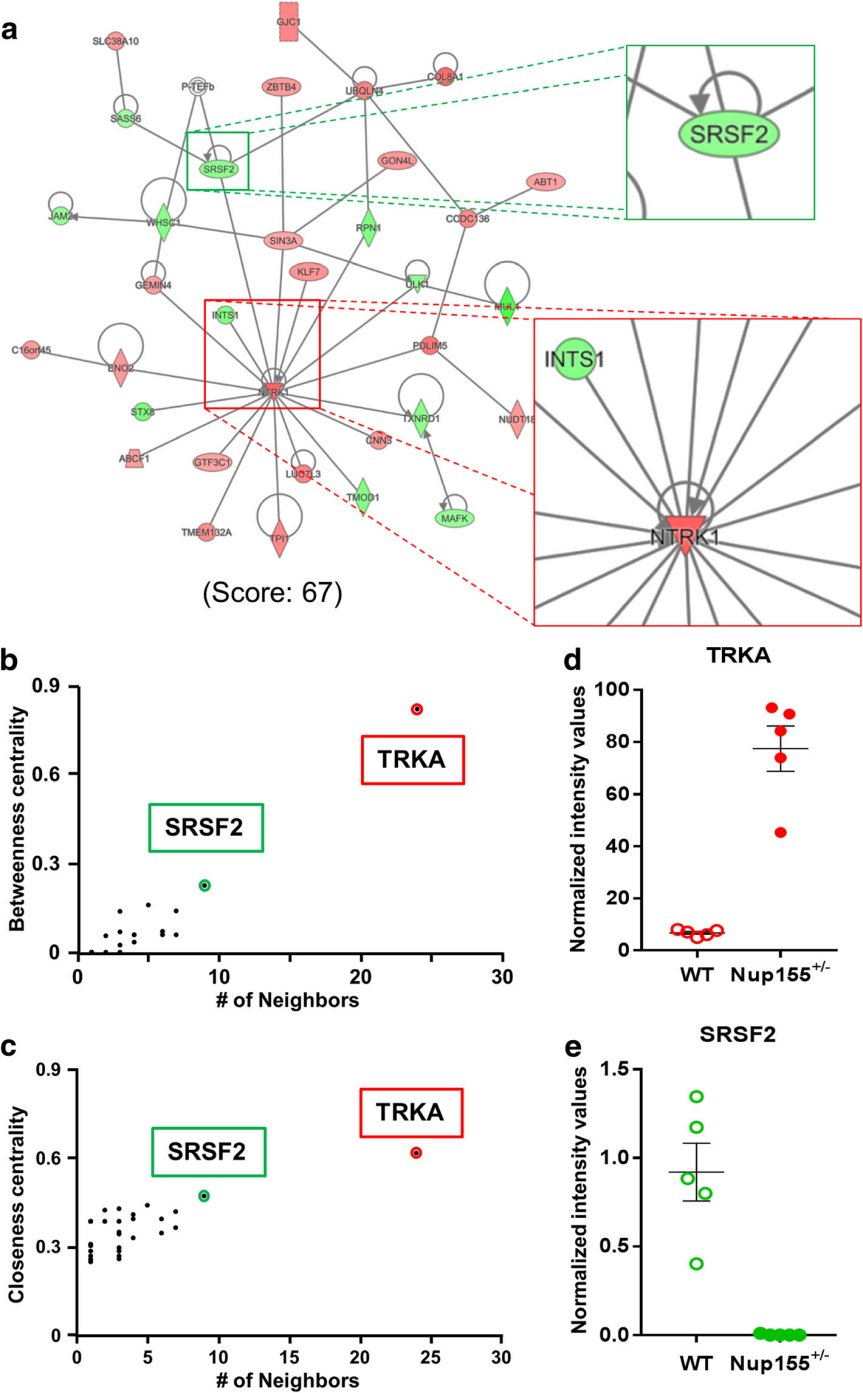
Deconvolution of modular functional enrichment within a sub-network of the NUP155^+/−^ transcriptome. **a** Sub-networks that comprise the larger network possess characteristic identities at the mesoscopic level. Identification of the most significant sub-network revealed robust and consistent functional enrichment in Cardiovascular System Development, and incorporated a variety of up and down-regulated genes. Magnification of hubs NTRK1/TRKA and SRSF2/SC35 shown on the right. **b**, **c** Betweenness and closeness centrality plots of this small network confirm the same molecule, NTRK1/TRKA, as critical for integration and information transmission within the module, labeled in both plots and highlighted in red. SRSF2/SC35 possessed the next highest betweenness and closeness centrality measures, labeled and highlighted in green. **d**, **e** RNAseq abundances for NTRK1/TRKA and SRSF2 confirmed significant expression changes for both transcripts. Normalized intensities shown on y-axis

Ingenuity Pathways Analysis reports molecular functional enrichment of each subnetwork within the collective network, and when complemented with DAVID-based analyses, comprehensive functional categories can be identified that one approach alone may not detect, as well as corroborate robust enrichment of consistent gene ontology categories [26–28]. This approach revealed the sub-network with the highest significance score (Score = 67) that prioritized Cardiovascular System Development and Function (Fig. 5a). Here, NTRK1/TRKA was integrated as the primary hub with the highest degree, betweenness and closeness centrality coefficients, followed by SRSF2/SC35. (Fig. 5b, c). Specific examination of NTRK1 and SRSF2 expression data in NUP155^+/−^ compared to WT control ES cell lines confirmed significance and magnitude of expression change (Fig. 5d, e). Western blot analysis (Additional file 1: Supplemental Methods) showed that SRSF2 and TRKA protein level changes followed the same trend as gene expression data, however densitometric analysis revealed no significant differences between NUP155^+/−^ and WT (Additional file 1: Figure S1).

## Discussion

A growing body of evidence supports multiple roles for nups in cell fate acquisition, yet the contributions of nups to normal differentiation are incompletely characterized. The present study employs a network strategy to deconvolute multivariate systems biology impacts of NUP155 in a pro-arrhythmogenic embryonic stem cell model of cardiogenesis and captures a capacity for NUP155 to remodel a pluripotent transcriptome. Key molecules associated with cardiac innervation and fibrosis were identified in a module enriched for Cardiovascular Development within a larger NUP155 remodeled network. This work identifies transcriptome recalibration caused by NUP155 deficiency that underlies arrhythmogenic elements, and supports a developmental function for nups beyond canonical roles in NPC architecture and nucleocytoplasmic transport.

To gain insights into allele frequencies of nups that may predispose to cardiac disease, we used the ExAC browser to investigate the anticipated number of variants for the nups we identified in our study, as well as their tolerance (or intolerance) to variation. All nups identified in the present work possess a high pLI score that is expected given the essential role of nucleoporins in eukaryotic cell viability. However, of the 5 identified in this study, *Nup85, Rae1,* and *Tpr* demonstrated a positive constraint metric z-score for missense mutations, while *Nup155* and *Nup153* had negative missense constraint z-scores. This suggests that *Nup155* and *Nup153* exist within the population with a high tolerance to variation that may present as developmental disease rather than terminal nonviability [30, 38]. Indeed, a landmark clinical study by Zhang et al. identified a mutation in NUP155 that led to atrial fibrillation [19], while recent work by Nanni et al. identified an altered role for NUP153 in cardiac chromatin regulation in patients with Duchenne muscular dystrophy [39]. In the former study, NUP155 protein levels were normal, with experimental models revealing disrupted nucleocytoplasmic transport that was concluded to be the main cause of arrhythmogenic compromise. The latter study identified a pathological up-regulation of NUP153 combined with increased NUP153 acetylation that led to dysregulated expression of nexilin with calcium channel gain of function [39]. It is of note that NUP155 regulates cardiac hypertrophy through HDAC4 [37], providing additional evidence for the potential role of nups as epigenomic regulators.

Indeed, the diversity of genes whose expression is dysregulated by NUP155 insufficiency suggests impacts on global mechanisms of gene regulation. In support of this, previous work has reported a capacity for NUP155 to bind, tether and regulate discrete regions of chromatin that control gene expression in yeast and *Drosophila* models [15, 40]. NUP155 may also act indirectly through interactions with histone modifiers, as described above for studies in rat models of cardiac hypertrophy [37], or inferred by electronic protein interaction datasets [41, 42]. In the present study, analysis of functional annotation clusters for both up and downregulated revealed consistent enrichment of terms related to membrane interactions and/or transmembrane biology. This was supported by non-clustered significant functional terms (Additional file 1: Tables S3 and S4). Select nucleoporins regulate cell adhesion primarily through altered nucleocytoplasmic trafficking [43], and NUP155 may influence cell membrane biology via this functional modality since NUP155 is critical for nuclear pore complex assembly, formation, and nuclear transport [13, 44, 45].

Controlled temporospatial execution of molecular programs is required for normal differentiation, driven by the composition and architecture of underlying gene networks. Disruption of these networks recalibrates pluripotency that leads to compromised phenotypes. In the present study, TP53 was prioritized in a molecular framework driven by NUP155 truncation, supported by recent demonstration of TP53 as a master regulator that controls hypertrophic responses of the myocardium [46]. Though investigation of our dataset revealed a non-significant increase in TP53 expression in heterozygous ES cell lines (data not shown), its prioritization in a pluripotent transcriptome network underlying cardiopathological manifestation is reinforced by recent systems biology meta-analyses of disease-causing protein-protein interaction (PPI) networks [47]. Pinero et al. describe in their study a general multiscale mesoscopic molecular signature that underlies disease, where tumor suppressors such as TP53 possess high centrality and are essential hubs within the network structure that are the most sensitive to genomic perturbation. The next prioritized network hubs are dominant disease genes, followed by recessive disease genes in modules with low centrality located at the network periphery [47]. Topology analysis of the present NUP155-recalibrated transcriptome is in line with this biological network property, as homozygous TP53 possesses the highest degree and closeness centrality scores that identify TP53 as a hub that integrates discrete network neighborhoods and determines informational flow among those regions.

NTRK1/TRKA and SRSF2/SC35 were identified here as the most upregulated and downregulated genes with highest degrees, respectively, within the sub-network that prioritizes Cardiovascular System Development, with mRNA and protein expression changes trending in the same direction. Although protein expression changes did not reach significance, such transcript and protein expression discrepancies are expected, given that multiple post-transcriptional and post-translational mechanisms may regulate final expression level [48]. This does not preclude the potential for NUP155 to impact cardiac development through a NUP155-TRKA signaling cascade, however. NTRK1/TRKA is a tyrosine receptor kinase that drives cholinergic differentiation [49], and has a defined role in promoting cardiac innervation and repair [50, 51]. NTRK1/TRKA directs target cardiac innervation through its effector, CORONIN-1 [52]. This developmental cascade is initiated upon upstream NGF stimulation of NTRK1/TRKA that drives CORONIN-1 mediated calcium release, which negatively regulates axon growth and arborization within the myocardium [52]. Increases in NTRK1/TRKA would facilitate CORONIN-1 suppression of cardiac innervation that would manifest as electrophysiological deficits. This is corroborated by studies that identify increased NTRK1/TRKA levels associated with atrial fibrillation, and is further supported by data that revealed concomitant autocrine and paracrine regulation of NTRK1/TRKA expression by upstream NGF [53]. Further work will clarify this potential NUP155-TRKA axis in the context of cardiac development.

The most downregulated hub identified within this Cardiovascular Development sub-network was SRSF2/SC35, implicated by protein expression data where the trend matched the decrease in SRSF2 mRNA. SRSF2 is a serine/arginine rich splicing factor essential for pluripotent self-renewal, with decreased SRSF2 promoting stem cell differentiation [54]. SRSF2 dynamics are highly regulated in cardiac tissue, as cardiac-specific ablation of SRSF2 results in dilated cardiomyopathy and abnormal Ca^2+^ handling linked to down-regulation of the cardiac specific ryanodine receptor [55]. Balanced interactions between SRSF2 and TBX5 are necessary for proper pre-mRNA splicing critical for cardiac development [56]. Indeed, disruptions to the TBX5/SRSF2 equilibrium result in Holt-Oram Syndrome, which include cardiac conduction diseases such as AF [57, 58] that can occur alone or in combination with atrial and ventricular septal defects [59].

## Conclusions

Gene networks are highly regulated, stratified structures that can be regulated at multiple levels [60]. Our results identify a mesoscopic cardiac sub-network impacted by NUP155-deficient recalibration of a pluripotent transcriptome. Here, NUP155 insufficiency re-organizes a molecular network that prioritizes TRKA and SRSF2 as potential factors in the development of cardiac AF. The idea that nups may epigenomically remodel the cardiac program is supported by the role of various nups in repressing and/or activating discrete chromatin regions [15, 40, 61–63] and previous work that identified regulated nup expression in cardiogenesis [26]. In particular, future work focused on elucidating the role of the splicing factor SRSF2 will provide deeper insights into the epigenomic function and cardiogenic role of NUP155 predicted by the present systems biology study.

## Additional file

Additional File 1: **Table S1.** Pathway Enrichment Analysis of Upregulated Genes. **Table S2.** Pathway Enrichment Analysis of Downregulated Genes. **Table S3.** Functional terms not clustered during pathway enrichment analysis of Upregulated genes. **Table S4.** Functional terms not clustered during pathway enrichment analysis of Downregulated genes. **Supplemental Methods**: Western Blot Analysis. **Figure S1.** Protein Expression data for SRSF2/SC35 and NTRK1/TRKA. Western blots of **a** SRSF2/SC35 and **b** NTRK1/TRKA from Wild Type (WT, *n* = 4) and Nup155+/− (*n =* 4) ES cell lines. β tubulin was used as loading control for both proteins. Comparison of normalized ratios from densitometry analyses of **c** SRSF2/SC35 and (D) NTRK1/TRKA protein levels in WT and Nup155+/− cell lines (*p* > 0.05). (PDF 416 kb)

## Abbreviations

5’ UTR: 5′ untranslated region
AF: Atrial fibrillation
APP: Amyloid beta precursor protein
Ca^2+^: Calcium
CM: Cardiomyocytes
CP: Cardiac precursors
CTNNB1: Beta catenin
DAVID: Database for annotation, visualization and integrated discovery
EBs: Embryoid bodies
ES: Embryonic stem
FBS: Fetal bovine serum
GEO: Gene expression omnibus
GTES: GMEM ES cell media
HNF4A: Hepatocyte nuclear factor 4 alpha
HOXA: Homeobox A cluster
LIF: Leukemia inhibitory factor
NDC1: Nuclear division cycle homolog 1
NEAA: Non-essential amino acids
NGF: Nerve growth factor
NHE1: Sodium/hydrogen exchanger 1
NPC: Nuclear pore complex
NTRK1/TRKA: Tropomyosin related kinase A
NUP: Nucleoporin
Nup155^+/−^: NUP155 exon trapped cell line
PBS: Phosphate buffered saline
QC: Quality control
RAE1: Ribonucleic acid export 1
ROI: Region of interest
SC35/SRSF2: Serine/arginine rich splicing factor 2
SOM: Self-organizing map
TBX5: T-box protein 5
TP53: Tumor protein 53
Tpr: Translocated promoter region
WT: Wild type
β-ME: Beta-mercaptoethanol
