# Supplementary material for: NUP155 insufficiency recalibrates a pluripotent transcriptome with network remodeling of a cardiogenic signaling module
Source: BMC Syst Biol. 2018 May 30;12:62. doi: 10.1186/s12918-018-0590-x (PMC5977756; doi:10.1186/s12918-018-0590-x)
Supplement: Supplementary file 1 — Table S1. Pathway Enrichment Analysis of Upregulated Genes. Table S2. Pathway Enrichment Analysis of Downregulated Genes. Table S3. Functional terms not clustered during pathway enrichment analysis of Upregulated genes. Table S4. Functional terms not clustered during pathway enrichment analysis of Downregulated genes. Supplemental Methods: Western Blot Analysis. Figure S1. Protein Expression data for SRSF2/SC35 and NTRK1/TRKA. Western blots of a SRSF2/SC35 and b NTRK1/TRKA from Wild Type (WT, n = 4) and Nup155+/− (n = 4) ES cell lines. β tubulin was used as loading control for both proteins. Comparison of normalized ratios from densitometry analyses of c SRSF2/SC35 and (D) NTRK1/TRKA protein levels in WT and Nup155+/− cell lines (p > 0.05). (PDF 416 kb) [file 12918_2018_590_MOESM1_ESM.pdf]

Supplemental Material.

Supplemental File 1.

Pathway Enrichment Analysis of Upregulated Genes.

Functional enrichment analysis was performed through DAVID using the highest stringency. Depicted below are the 14 functional clusters extracted from the statistically significant upregulated gene list.

Cluster 1	Category	Term	Count	%	Entre Gene IDs	PValue	Fold Enrichment
Enrichment Score: 1.84	UP_SEQ_FEATURE	short sequence motif:GFFKR motif	3	1.705	16402, 381924, 16399	0.005	27.897
	INTERPRO	IPR018184:Integrin alpha chain, C-terminal cytoplasmic region, conserved site	3	1.705		0.007	22.714
	INTERPRO	IPR000413:Integrin alpha chain	3	1.705		0.008	21.452
	INTERPRO	IPR013649:Integrin alpha-2	3	1.705		0.008	21.452
	INTERPRO	IPR013517:FG-GAP repeat	3	1.705		0.008	21.452
	UP_SEQ_FEATURE	repeat:FG-GAP 7	3	1.705		0.008	21.333
	UP_SEQ_FEATURE	repeat:FG-GAP 5	3	1.705		0.008	21.333
	UP_SEQ_FEATURE	repeat:FG-GAP 6	3	1.705		0.008	21.333
	UP_SEQ_FEATURE	repeat:FG-GAP 3	3	1.705		0.008	21.333
	UP_SEQ_FEATURE	repeat:FG-GAP 4	3	1.705		0.008	21.333
	UP_SEQ_FEATURE	repeat:FG-GAP 1	3	1.705		0.008	21.333
	UP_SEQ_FEATURE	repeat:FG-GAP 2	3	1.705		0.008	21.333
	INTERPRO	IPR013519:Integrin alpha beta-propellor	3	1.705		0.009	20.323
	SMART	SM00191:Int_alpha	3	1.705		0.012	17.511
	GOTERM_CC_DIRECT	GO:0008305~integrin complex	3	1.705		0.016	15.321
	UP_KEYWORDS	Integrin	3	1.705		0.059	7.596
	GOTERM_BP_DIRECT	GO:0007229~integrin-mediated signaling pathway	3	1.705		0.168	4.051
	KEGG_PATHWAY	mmu04810:Regulation of actin cytoskeleton	3	1.705		0.550	1.615
Cluster 2							
Enrichment Score: 1.50	Category	Term	Count	%	Entre Gene IDs	PValue	Fold Enrichment
	INTERPRO	IPR000719:Protein kinase, catalytic domain	10	5.682	18752, 17347, 54721, 18211, 18647, 83813, 18214, 269224, 18759, 11790	0.019	2.499
	GOTERM_MF_DIRECT	GO:0004672~protein kinase activity	10	5.682		0.029	2.314
	INTERPRO	IPR011009:Protein kinase-like domain	10	5.682		0.029	2.315
	GOTERM_BP_DIRECT	GO:0006468~protein phosphorylation	10	5.682		0.040	2.180
	GOTERM_BP_DIRECT	GO:0016310~phosphorylation	10	5.682		0.054	2.052
Cluster 3	Category	Term	Count	%	Entre Gene IDs	PValue	Fold Enrichment
Enrichment Score: 1.35	INTERPRO	IPR020635:Tyrosine-protein kinase, catalytic domain	4	2.273	54721, 18211, 83813, 18214	0.025	6.356
	SMART	SM00219:TyrKc	4	2.273		0.036	5.477
	INTERPRO	IPR008266:Tyrosine-protein kinase, active site	4	2.273		0.041	5.201
	GOTERM_BP_DIRECT	GO:0007169~transmembrane receptor protein tyrosine kinase signaling pathway	4	2.273		0.045	5.023
	UP_KEYWORDS	Tyrosine-protein kinase	4	2.273		0.052	4.750
	INTERPRO	IPR001245:Serine-threonine/tyrosine-protein kinase catalytic domain	4	2.273		0.093	3.704
Cluster 4	Category	Term	Count	%	Entre Gene IDs	PValue	Fold Enrichment
Enrichment Score: 0.89	INTERPRO	IPR008160:Collagen triple helix repeat	3	1.705	140792, 12842, 12837	0.117	5.081
	UP_KEYWORDS	Collagen	3	1.705		0.131	4.737
	GOTERM_CC_DIRECT	GO:0005581~collagen trimer	3	1.705		0.136	4.615
Cluster 5	Category	Term	Count	%	Entre Gene IDs	PValue	Fold Enrichment
Enrichment Score: 0.74	UP_SEQ_FEATURE	domain:EGF-like 4	3	1.705	17183, 73182, 224024	0.075	6.594
	UP_SEQ_FEATURE	domain:EGF-like 3	3	1.705		0.102	5.495
	UP_SEQ_FEATURE	domain:EGF-like 2	3	1.705		0.152	4.317
	UP_SEQ_FEATURE	domain:EGF-like 1	3	1.705		0.232	3.267
	INTERPRO	IPR009030:Insulin-like growth factor binding protein, N-terminal	3	1.705		0.266	2.970
	SMART	SM00181:EGF	3	1.705		0.482	1.838
Cluster 6	Category	Term	Count	%	Entre Gene IDs	PValue	Fold Enrichment
Enrichment Score: 0.71	INTERPRO	IPR008271:Serine/threonine-protein kinase, active site	6	3.409	18752, 17347, 18647, 269224, 18759, 11790	0.116	2.319
	UP_KEYWORDS	Serine/threonine-protein kinase	6	3.409		0.183	1.988
	SMART	SM00220:S_TKc	6	3.409		0.251	1.751
	GOTERM_MF_DIRECT	GO:0004674~protein serine/threonine kinase activity	6	3.409		0.265	1.722
Cluster 7	Category	Term	Count	%	Entre Gene IDs	PValue	Fold Enrichment
Enrichment Score: 0.64	UP_SEQ_FEATURE	metal ion-binding site:Zinc; in inhibited form	3	1.705	11504, 17388, 23794	0.024	12.505
	UP_SEQ_FEATURE	short sequence motif:Cysteine switch	3	1.705		0.041	9.299
	INTERPRO	IPR024079:Metallopeptidase, catalytic domain	3	1.705		0.172	3.981
	GOTERM_MF_DIRECT	GO:0004222~metalloendopeptidase activity	3	1.705		0.262	2.997
	UP_KEYWORDS	Metalloprotease	3	1.705		0.284	2.815
	GOTERM_MF_DIRECT	GO:0008237~metallopeptidase activity	3	1.705		0.372	2.304
	UP_KEYWORDS	Zymogen	3	1.705		0.475	1.890
	UP_KEYWORDS	Protease	3	1.705		0.913	0.743
	GOTERM_MF_DIRECT	GO:0008233~peptidase activity	3	1.705		0.924	0.714
Cluster 8	Category	Term	Count	%	Entre Gene IDs	PValue	Fold Enrichment
Enrichment Score: 0.34	INTERPRO	IPR001806:Small GTPase superfamily	3	1.705	19340, 19338, 19416	0.277	2.882
	UP_KEYWORDS	Prenylation	3	1.705		0.322	2.564
	INTERPRO	IPR005225:Small GTP-binding protein domain	3	1.705		0.366	2.340
	GOTERM_BP_DIRECT	GO:0007264~small GTPase mediated signal transduction	3	1.705		0.559	1.596
	UP_KEYWORDS	GTP-binding	3	1.705		0.708	1.213
	UP_SEQ_FEATURE	nucleotide phosphate-binding region:GTP	3	1.705		0.741	1.137

Supplemental File 1. Continuation.

Cluster 9	Category	Term	Count	%	Entre Gene IDs	PValue	Fold Enrichment
Enrichment Score: 0.28	UP_SEQ_FEATURE	repeat:ANK 1	3	1.705	142688, 320150, 71452	0.469	1.879
	UP_SEQ_FEATURE	repeat:ANK 2	3	1.705		0.469	1.879
	UP_KEYWORDS	ANK repeat	3	1.705		0.533	1.678
	INTERPRO	IPR002110:Ankyrin repeat	3	1.705		0.546	1.664
	INTERPRO	IPR020683:Ankyrin repeat-containing domain	3	1.705		0.560	1.596
	SMART	SM00248:ANK	3	1.705		0.600	1.479

Cluster 10	Category	Term	Count	%	Entre Gene IDs	PValue	Fold Enrichment
Enrichment Score: 0.21	UP_KEYWORDS	mRNA splicing	3	1.705	67959, 67684, 75956	0.533	1.678
	GOTERM_BP_DIRECT	GO:0008380~RNA splicing	3	1.705		0.571	1.563
	UP_KEYWORDS	mRNA processing	3	1.705		0.666	1.311
	GOTERM_BP_DIRECT	GO:0006397~mRNA processing	3	1.705		0.726	1.170

Cluster 11	Category	Term	Count	%	Entre Gene IDs	PValue	Fold Enrichment
Enrichment Score: 0.19	UP_KEYWORDS	Homeobox	3	1.705	21410, 384452, 210719	0.616	1.438
	INTERPRO	IPR001356:Homeodomain	3	1.705		0.621	1.425
	SMART	SM00389:HOX	3	1.705		0.691	1.251

Cluster 12	Category	Term	Count	%	Entre Gene IDs	PValue	Fold Enrichment
Enrichment Score: 0.18	UP_KEYWORDS	Transmembrane helix	51	28.977	70310, 11539, 233863, 224024, 217219, 16402, 72055, 207683, 17388, 72555, 320150, 12723, 14615, 243168, 100043125, 54420, 380712, 12223, 232664, 12794, 18211, 170813, 18214, 12558, 20969, 17355, 218271, 50918, 60504, 11552, 381924, 228368, 75571, 258595, 20452, 13642, 16399, 14764, 18667, 241116, 382551, 98170, 77254, 12737, 54445, 15937, 73182, 140792, 239845, 20392, 14362	0.621	0.986
	UP_KEYWORDS	Transmembrane	51	28.977		0.629	0.984
	GOTERM_CC_DIRECT	GO:0016021~integral component of membrane	51	28.977		0.752	0.947

Cluster 13	Category	Term	Count	%	Entre Gene IDs	PValue	Fold Enrichment
Enrichment Score: 0.13	INTERPRO	IPR003598:Immunoglobulin subtype 2	3	1.705	98733, 207683, 11790	0.560	1.596
	SMART	SM00408:IGc2	3	1.705	98733, 207683, 11790	0.640	1.375
	INTERPRO	IPR003599:Immunoglobulin subtype	3	1.705	98733, 207683, 11790	0.912	0.745
	SMART	SM00409:IG	3	1.705	98733, 207683, 11790	0.949	0.642

Cluster 14	Category	Term	Count	%	Entre Gene IDs	PValue	Fold Enrichment
Enrichment Score: 0.04	INTERPRO	IPR013087:Zinc finger C2H2-type/integrase DNA-binding domain	4	2.273	56525, 93691, 230761, 75580	0.883	0.790
	INTERPRO	IPR015880:Zinc finger, C2H2-like	4	2.273	56525, 93691, 230761, 75580	0.907	0.743
	INTERPRO	IPR007087:Zinc finger, C2H2	4	2.273	56525, 93691, 230761, 75580	0.925	0.704
	SMART	SM00355:ZnF_C2H2	4	2.273	56525, 93691, 230761, 75580	0.952	0.640

Supplemental File 2.

Pathway Enrichment Analysis of Downregulated Genes

Functional enrichment analysis was performed through DAVID using the highest stringency. Depicted below are the 11 functional clusters extracted from the statistically significant downregulated gene list.

Cluster 1	Category	Term	Count	%	Genes	PValue	Fold Enrichment
Enrichment Score: 1.55	INTERPRO	IPR013087:Zinc finger C2H2-type/integrase DNA-binding domain	10	6.67	110593, 112415, 56490, 328274, 100233208, 100041433, 70081, 433791, 433520, 17764	0.019	2.487
	INTERPRO	IPR015880:Zinc finger, C2H2-like	10	6.67		0.027	2.340
	SMART	SM00355:ZnF_C2H2	10	6.67		0.045	2.089

Cluster 2	Category	Term	Count	%	Genes	PValue	Fold Enrichment
Enrichment Score: 0.90	GOTERM_BP_DIRECT	GO:0018108~peptidyl-tyrosine phosphorylation	3	2.00	14254, 13838, 14360	0.069	6.897
	INTERPRO	IPR020635:Tyrosine-protein kinase, catalytic domain	3	2.00		0.088	6.006
	SMART	SM00219:TyrKc	3	2.00		0.105	5.363
	INTERPRO	IPR008266:Tyrosine-protein kinase, active site	3	2.00		0.123	4.914
	UP_KEYWORDS	Tyrosine-protein kinase	3	2.00		0.147	4.395
	GOTERM_MF_DIRECT	GO:0004713~protein tyrosine kinase activity	3	2.00		0.197	3.635
	INTERPRO	IPR001245:Serine-threonine/tyrosine-protein kinase catalytic domain	3	2.00		0.209	3.500

Cluster 3	Category	Term	Count	%	Genes	PValue	Fold Enrichment
Enrichment Score: 0.59	UP_SEQ_FEATURE	calcium-binding region:2	3	2.00	18570, 170638, 13518	0.160	4.158
	UP_SEQ_FEATURE	calcium-binding region:1	3	2.00		0.187	3.762
	SMART	SM00054:EFh	3	2.00		0.260	2.996
	INTERPRO	IPR018247:EF-Hand 1, calcium-binding site	3	2.00		0.290	2.780
	UP_SEQ_FEATURE	domain:EF-hand 2	3	2.00		0.296	2.740
	UP_SEQ_FEATURE	domain:EF-hand 1	3	2.00		0.298	2.724
	INTERPRO	IPR002048:EF-hand domain	3	2.00		0.397	2.181

Cluster 4	Category	Term	Count	%	Genes	PValue	Fold Enrichment
Enrichment Score: 0.56	INTERPRO	IPR000719:Protein kinase, catalytic domain	6	4.00	14254, 13838, 27263, 24013, 14360, 22241	0.208	1.889
	GOTERM_MF_DIRECT	GO:0004672~protein kinase activity	6	4.00		0.290	1.657
	GOTERM_BP_DIRECT	GO:0006468~protein phosphorylation	6	4.00		0.336	1.557

Cluster 5	Category	Term	Count	%	Genes	PValue	Fold Enrichment
Enrichment Score: 0.50	UP_SEQ_FEATURE	repeat:ANK 5	3	2.00	67434, 329251, 142687	0.147	4.389
	UP_SEQ_FEATURE	repeat:ANK 4	3	2.00		0.190	3.732
	UP_SEQ_FEATURE	repeat:ANK 3	3	2.00		0.266	2.963
	UP_SEQ_FEATURE	repeat:ANK 1	3	2.00		0.342	2.456
	UP_SEQ_FEATURE	repeat:ANK 2	3	2.00		0.342	2.456
	INTERPRO	IPR002110:Ankyrin repeat	3	2.00		0.416	2.097
	UP_KEYWORDS	ANK repeat	3	2.00		0.423	2.069
	INTERPRO	IPR020683:Ankyrin repeat-containing domain	3	2.00		0.437	2.010
	SMART	SM00248:ANK	3	2.00		0.455	1.931

Cluster 6	Category	Term	Count	%	Genes	PValue	Fold Enrichment
Enrichment Score: 0.50	UP_SEQ_FEATURE	repeat:3	3	2.00	72685, 16549, 71844	0.242	3.160
	UP_SEQ_FEATURE	repeat:1	3	2.00		0.358	2.370
	UP_SEQ_FEATURE	repeat:2	3	2.00		0.365	2.335

Cluster 7	Category	Term	Count	%	Genes	PValue	Fold Enrichment
Enrichment Score: 0.44	UP_SEQ_FEATURE	domain:BTB	3	2.00	56490, 20747, 75785	0.240	3.181
	INTERPRO	IPR000210:BTB/POZ-like	3	2.00		0.395	2.191
	INTERPRO	IPR011333:BTB/POZ fold	3	2.00		0.416	2.097
	SMART	SM00225:BTB	3	2.00		0.439	1.993

Cluster 8	Category	Term	Count	%	Genes	PValue	Fold Enrichment
Enrichment Score: 0.33	UP_KEYWORDS	Immunoglobulin domain	5	3.33	74048, 14254, 12519, 106347, 67374	0.326	1.721
	INTERPRO	IPR003599:Immunoglobulin subtype	5	3.33		0.391	1.565
	SMART	SM00409:IG	5	3.33		0.473	1.398
	INTERPRO	IPR007110:Immunoglobulin-like domain	5	3.33		0.820	0.881

Cluster 9	Category	Term	Count	%	Genes	PValue	Fold Enrichment
Enrichment Score: 0.06	UP_KEYWORDS	Protease	3	2.00	215351, 214523, 73382	0.839	0.916
	GOTERM_MF_DIRECT	GO:0008233~peptidase activity	3	2.00		0.868	0.852
	GOTERM_BP_DIRECT	GO:0006508~proteolysis	3	2.00		0.902	0.770

Cluster 10	Category	Term	Count	%	Genes	PValue	Fold Enrichment
Enrichment Score: 0.05	UP_KEYWORDS	Transcription regulation	9	6.00	15424, 107823, 16549, 56490, 70461, 74042, 20182, 17764, 17135	0.854	0.828
	UP_KEYWORDS	Transcription	9	6.00		0.877	0.801
	GOTERM_BP_DIRECT	GO:0006351~transcription, DNA-templated	9	6.00		0.941	0.713

Cluster 11	Category	Term	Count	%	Genes	PValue	Fold Enrichment
Enrichment Score: 0.002	UP_KEYWORDS	Olfaction	3	2.00	259111, 258713, 258604	0.993	0.429
	INTERPRO	IPR000725:Olfactory receptor	3	2.00		0.994	0.423
	KEGG_PATHWAY	mmu04740:Olfactory transduction	3	2.00		0.996	0.412
	GOTERM_BP_DIRECT	GO:0007608~sensory perception of smell	3	2.00		0.996	0.392
	GOTERM_MF_DIRECT	GO:0004984~olfactory receptor activity	3	2.00		0.997	0.381

Supplemental File 3.
Functional terms not clustered during pathway enrichment analysis of Upregulated genes.

Category	Term	Count	%	Genes	PValue	Fold Enrichment
GOTERM_MF_DIRECT	**GO:0005515~protein binding**	55	31.25	17347, 20437, 72836, 11539, 67254, 16890, 16402, 140917, 320150, 19340, 14615, 54420, 15957, 212706, 21770, 384783, 432611, 13385, 27418, 18211, 83409, 170813, 11766, 12558, 56390, 76742, 21410, 53310, 17826, 17192, 12703, 18667, 76022, 382551, 244059, 12394, 13435, 244058, 12842, 56376, 14260, 12737, 16882, 18759, 210719, 20466, 19338, 15937, 18752, 67220, 11504, 71994, 20392, 15519, 22628	0.0001	1.651
UP_KEYWORDS	**Phosphoprotein**	82	46.59	17313, 17347, 80288, 68166, 75956, 20437, 233863, 224024, 13998, 16890, 94232, 14432, 18647, 269224, 217219, 16402, 72055, 12723, 76813, 224742, 243168, 230793, 54721, 15957, 212706, 18739, 13807, 12349, 232664, 384783, 13385, 18211, 214459, 98733, 83813, 18214, 20969, 56390, 17355, 75580, 67684, 76742, 11790, 13642, 21410, 114601, 53310, 17826, 17192, 195434, 18667, 14764, 67959, 12350, 76022, 13435, 244059, 12394, 208177, 329739, 77254, 12842, 140486, 56376, 14260, 16592, 16882, 18759, 71452, 105203, 54445, 20466, 15937, 67220, 53414, 18752, 73182, 17974, 76156, 21991, 22628, 15519	0.0001	1.445
GOTERM_CC_DIRECT	**GO:0016020~membrane**	77	43.75	68166, 70310, 11539, 233863, 224024, 16890, 14432, 18647, 217219, 16402, 17388, 207683, 72055, 72555, 320150, 12723, 224742, 19340, 14615, 19416, 54721, 54420, 380712, 212706, 18739, 13807, 12223, 12349, 232664, 13385, 12794, 18211, 83409, 214459, 83813, 18214, 170813, 11766, 12558, 20969, 252903, 218271, 50918, 60504, 76742, 381924, 228368, 75571, 13642, 20452, 276919, 114601, 16399, 14764, 241116, 382551, 98170, 244058, 77254, 208177, 56376, 14260, 12737, 18759, 54445, 15937, 19338, 67220, 53414, 18752, 140792, 73182, 239845, 20392, 22628, 15519, 14362	0.0002	1.405
UP_SEQ_FEATURE	**compositionally biased region:Glu-rich**	10	5.68	17192, 73884, 76022, 241116, 67684, 73569, 232664, 114601, 224742, 432611	0.0003	4.614
GOTERM_CC_DIRECT	**GO:0014069~postsynaptic density**	9	5.11	18752, 14432, 56376, 71994, 17974, 11539, 13385, 12794, 53310	0.0006	4.808
GOTERM_CC_DIRECT	**GO:0045202~synapse**	13	7.39	56376, 11539, 12794, 13385, 53310, 18752, 239845, 14432, 16402, 12558, 320150, 72555, 17974	0.0006	3.287
UP_KEYWORDS	**Methylation**	18	10.23	230793, 80288, 18739, 75956, 11790, 13642, 384783, 19338, 53414, 406220, 71994, 207683, 21991, 68524, 13435, 12394, 19340, 19416	0.0008	2.516
UP_KEYWORDS	**Alternative splicing**	54	30.68	17347, 80288, 68166, 75956, 233863, 13998, 16890, 18647, 217219, 140917, 72055, 72555, 320150, 76813, 233001, 243168, 380712, 232664, 432611, 56812, 13385, 213484, 98733, 83813, 214459, 20969, 218271, 105428, 67684, 76742, 75571, 11790, 21410, 114601, 17826, 17192, 67959, 12394, 244058, 353282, 208177, 77254, 12842, 56376, 14260, 16882, 71452, 54445, 20466, 17183, 73182, 239170, 76156, 20392	0.0009	1.516
GOTERM_CC_DIRECT	**GO:0005925~focal adhesion**	11	6.25	208177, 18214, 16402, 71994, 12558, 20969, 13642, 22628, 224024, 16399, 14362	0.0010	3.592
GOTERM_CC_DIRECT	**GO:0032281~AMPA glutamate receptor complex**	4	2.27	72555, 13385, 12794, 53310	0.0016	17.023
GOTERM_CC_DIRECT	**GO:0030659~cytoplasmic vesicle membrane**	6	3.41	68166, 212706, 11766, 320150, 252903, 22628	0.0025	6.384
GOTERM_BP_DIRECT	**GO:0042220~response to cocaine**	4	2.27	18667, 15519, 13435, 13385	0.0033	13.218
UP_KEYWORDS	**Cell junction**	13	7.39	56376, 54420, 14260, 12737, 12794, 13385, 18752, 239845, 14432, 16402, 320150, 72555, 14615	0.0039	2.639
GOTERM_CC_DIRECT	**GO:0030054~cell junction**	14	7.95	56376, 54420, 14260, 12737, 12794, 13385, 18752, 239845, 14432, 67959, 16402, 72555, 320150, 14615	0.0041	2.489
GOTERM_CC_DIRECT	**GO:0031410~cytoplasmic vesicle**	13	7.39	212706, 68166, 54445, 12703, 94232, 18211, 214459, 11504, 16402, 11766, 320150, 252903, 109685	0.0047	2.569
GOTERM_CC_DIRECT	**GO:0016323~basolateral plasma membrane**	7	3.98	54420, 12558, 12349, 11539, 15519, 13385, 53310	0.0052	4.403
GOTERM_MF_DIRECT	**GO:0019903~protein phosphatase binding**	5	2.84	12558, 15519, 384783, 13385, 53310	0.0056	6.981
GOTERM_CC_DIRECT	**GO:0005737~cytoplasm**	68	38.64	17347, 227290, 68166, 20437, 70827, 13998, 16890, 94232, 14432, 18647, 269224, 16402, 140917, 320150, 233001, 224742, 19416, 54721, 15957, 18739, 13807, 12349, 384783, 13385, 432611, 66089, 27418, 18211, 98733, 83813, 214459, 170813, 12558, 20969, 76742, 276919, 105663, 18767, 53310, 17826, 17192, 12703, 18667, 12350, 68524, 12394, 13435, 208177, 12842, 140486, 56376, 14260, 16592, 12737, 18759, 105203, 20466, 67220, 53414, 18752, 11504, 71994, 76156, 17974, 20392, 15519, 22628, 73047	0.0059	1.309
GOTERM_CC_DIRECT	**GO:0043197~dendritic spine**	6	3.41	18667, 140486, 71994, 11539, 13385, 12794	0.0061	5.176
GOTERM_CC_DIRECT	**GO:0031012~extracellular matrix**	8	4.55	218271, 17313, 12842, 11504, 17388, 23794, 15519, 12837	0.0083	3.474
UP_SEQ_FEATURE	**topological domain:Extracellular**	30	17.05	60504, 11552, 70310, 381924, 11539, 13642, 224024, 16399, 14764, 382551, 16402, 217219, 17388, 207683, 98170, 14615, 77254, 54420, 12737, 12223, 15937, 140792, 239845, 18211, 170813, 18214, 12558, 20969, 20392, 14362	0.0092	1.608
INTERPRO	**IPR017441:Protein kinase, ATP binding site**	9	5.11	18752, 17347, 54721, 18211, 18647, 83813, 269224, 18759, 11790	0.0116	2.940
UP_SEQ_FEATURE	**active site:Proton acceptor**	13	7.39	243168, 54721, 17347, 13807, 11790, 18759, 18752, 18211, 18647, 83813, 269224, 18214, 21991	0.0143	2.213
GOTERM_BP_DIRECT	**GO:0046777~protein autophosphorylation**	6	3.41	18752, 17347, 18211, 83813, 18214, 269224	0.0152	4.117
GOTERM_MF_DIRECT	**GO:0004713~protein tyrosine kinase activity**	5	2.84	54721, 18211, 83813, 18214, 15519	0.0168	5.077
UP_SEQ_FEATURE	**metal ion-binding site:Zinc; catalytic**	5	2.84	12350, 11504, 17388, 23794, 12349	0.0168	5.079
UP_KEYWORDS	**Synapse**	8	4.55	18752, 14432, 239845, 56376, 72555, 320150, 13385, 12794	0.0173	3.007
GOTERM_BP_DIRECT	**GO:0016477~cell migration**	6	3.41	54721, 12558, 17974, 20969, 18759, 13642	0.0180	3.945
GOTERM_BP_DIRECT	**GO:0035987~endodermal cell differentiation**	3	1.70	16402, 17388, 12837	0.0192	13.952
UP_SEQ_FEATURE	**compositionally biased region:Poly-Ser**	9	5.11	12703, 239845, 18739, 75571, 30946, 11790, 384783, 17355, 56812	0.0194	2.673
UP_SEQ_FEATURE	**binding site:ATP**	11	6.25	18752, 17347, 54721, 18211, 18647, 83813, 18214, 269224, 16882, 18759, 11790	0.0223	2.281
GOTERM_BP_DIRECT	**GO:0010976~positive regulation of neuron projection development**	5	2.84	18211, 21770, 11504, 18759, 244058	0.0240	4.550
UP_SEQ_FEATURE	**compositionally biased region:Cys-rich**	5	2.84	73884, 70310, 11504, 23794, 20392	0.0253	4.477
UP_KEYWORDS	**Palmitate**	7	3.98	14432, 18667, 11552, 70310, 320150, 11539, 13385	0.0262	3.090
GOTERM_CC_DIRECT	**GO:0097060~synaptic membrane**	3	1.70	18752, 13807, 13385	0.0271	11.607
UP_KEYWORDS	**Cytoplasmic vesicle**	9	5.11	12703, 94232, 214459, 68166, 212706, 11766, 320150, 252903, 54445	0.0296	2.470
GOTERM_CC_DIRECT	**GO:0045211~postsynaptic membrane**	6	3.41	239845, 56376, 11539, 13385, 12794, 53310	0.0300	3.451
GOTERM_MF_DIRECT	**GO:0042802~identical protein binding**	11	6.25	12842, 94232, 54420, 67959, 20437, 12737, 320150, 56390, 15519, 13435, 16399	0.0305	2.162
KEGG_PATHWAY	**mmu04910:Insulin signaling pathway**	5	2.84	17347, 12703, 16890, 18759, 384783	0.0314	4.115
UP_SEQ_FEATURE	**splice variant**	50	28.41	17347, 80288, 68166, 75956, 233863, 13998, 217219, 140917, 72055, 320150, 76813, 233001, 243168, 380712, 232664, 432611, 56812, 13385, 213484, 83813, 214459, 20969, 218271, 105428, 67684, 76742, 75571, 11790, 21410, 114601, 17192, 67959, 12394, 13435, 244058, 353282, 208177, 77254, 12842, 56376, 14260, 16882, 71452, 54445, 20466, 17183, 73182, 239170, 76156, 20392	0.0329	1.288
GOTERM_BP_DIRECT	**GO:0006605~protein targeting**	3	1.70	252903, 22628, 70827	0.0363	9.913
GOTERM_CC_DIRECT	**GO:0005829~cytosol**	22	12.50	56376, 76742, 68166, 20437, 12349, 18759, 384783, 276919, 56812, 27418, 18752, 16890, 12703, 18667, 94232, 12350, 83813, 18647, 21991, 22628, 15519, 73047	0.0364	1.574
GOTERM_BP_DIRECT	**GO:0030199~collagen fibril organization**	3	1.70	12842, 18214, 210719	0.0380	9.659

Supplemental File 3. Continuation.

Category	Term	Count	%	Genes	PValue	Fold Enrichment
GOTERM_CC_DIRECT	**GO:0030425~dendrite**	9	5.11	18752, 18211, 18667, 140486, 71994, 11539, 20392, 13385, 12794	0.0381	2.345
UP_KEYWORDS	**Kinase**	11	6.25	18752, 17347, 54721, 18211, 18647, 83813, 18214, 269224, 18759, 11790, 73047	0.0381	2.088
UP_KEYWORDS	**Membrane**	77	43.75	68166, 70310, 11539, 233863, 224024, 16890, 14432, 18647, 217219, 16402, 17388, 207683, 72055, 72555, 320150, 12723, 19340, 14615, 19416, 243168, 100043125, 54420, 380712, 212706, 18739, 13807, 12223, 12349, 232664, 13385, 12794, 18211, 83409, 214459, 83813, 18214, 170813, 11766, 12558, 20969, 252903, 17355, 218271, 50918, 60504, 11552, 76742, 381924, 258595, 228368, 75571, 13642, 20452, 16399, 18667, 14764, 241116, 382551, 98170, 244058, 77254, 208177, 56376, 14260, 12737, 18759, 54445, 15937, 19338, 67220, 18752, 73182, 140792, 239845, 20392, 15519, 14362	0.0383	1.190
GOTERM_CC_DIRECT	**GO:0005886~plasma membrane**	49	27.84	50918, 11552, 68166, 70310, 20437, 258595, 11539, 13642, 70827, 53310, 16890, 18667, 14764, 14432, 241116, 18647, 382551, 16402, 207683, 72555, 320150, 12723, 19340, 244058, 14615, 19416, 54420, 56376, 14260, 12737, 13807, 12223, 12349, 18759, 384783, 13385, 12794, 67220, 18752, 73182, 239845, 18211, 214459, 18214, 12558, 20392, 15519, 14362	0.0384	1.284
GOTERM_BP_DIRECT	**GO:0007155~cell adhesion**	9	5.11	53414, 16402, 381924, 207683, 12558, 13642, 224024, 16399, 12837	0.0391	2.330
KEGG_PATHWAY	**mmu04512:ECM-receptor interaction**	4	2.27	12842, 16402, 20969, 16399	0.0391	5.237
UP_KEYWORDS	**Cell membrane**	38	21.59	11552, 68166, 258595, 11539, 16890, 14432, 14764, 241116, 18647, 382551, 207683, 72555, 320150, 12723, 19340, 244058, 14615, 19416, 54420, 56376, 14260, 12737, 13807, 12223, 12349, 13385, 12794, 18752, 67220, 73182, 239845, 18211, 214459, 18214, 12558, 20392, 15519, 14362	0.0393	1.357
KEGG_PATHWAY	**mmu04390:Hippo signaling pathway**	5	2.84	18759, 22628, 13385, 14362, 53310	0.0398	3.815
UP_SEQ_FEATURE	**topological domain:Cytoplasmic**	33	18.75	218271, 60504, 11552, 70310, 381924, 11539, 13642, 20452, 224024, 16399, 14764, 382551, 16402, 217219, 207683, 17388, 320150, 12723, 98170, 14615, 77254, 54420, 12737, 12223, 15937, 140792, 239845, 170813, 18214, 12558, 20969, 20392, 14362	0.0427	1.385
UP_KEYWORDS	**Calcium**	12	6.82	17313, 18752, 140792, 12842, 70310, 16402, 18739, 381924, 11504, 17388, 12558, 16399	0.0442	1.947
UP_KEYWORDS	**Cytoplasm**	43	24.43	17347, 76742, 68166, 20437, 105663, 13998, 17826, 16890, 18667, 94232, 12350, 18647, 269224, 140917, 68524, 13435, 233001, 224742, 19416, 208177, 140486, 15957, 56376, 18739, 14260, 16592, 13807, 18759, 12349, 384783, 432611, 27418, 18752, 53414, 67220, 98733, 83813, 214459, 17974, 20392, 15519, 22628, 73047	0.0442	1.310
GOTERM_BP_DIRECT	**GO:0032355~response to estradiol**	4	2.27	17192, 12842, 17388, 13435	0.0461	4.973
GOTERM_CC_DIRECT	**GO:0043005~neuron projection**	8	4.55	18752, 14764, 18667, 56376, 13807, 15519, 244058, 14362	0.0463	2.432
GOTERM_MF_DIRECT	**GO:0016301~kinase activity**	11	6.25	18752, 17347, 54721, 18211, 18647, 83813, 18214, 269224, 18759, 11790, 73047	0.0469	2.005
GOTERM_CC_DIRECT	**GO:0045171~intercellular bridge**	3	1.70	227290, 18759, 20466	0.0479	8.512
GOTERM_CC_DIRECT	GO:0042995~cell projection	11	6.25	27418, 18752, 53414, 14432, 241116, 140486, 72555, 20392, 13385, 12794, 432611	0.0518	1.973
KEGG_PATHWAY	mmu05202:Transcriptional misregulation in cancer	5	2.84	18211, 18647, 17355, 12394, 20466	0.0531	3.471
UP_KEYWORDS	Golgi apparatus	11	6.25	218271, 98733, 18739, 228368, 11766, 320150, 20392, 20452, 252903, 98170, 19338	0.0541	1.960
UP_SEQ_FEATURE	compositionally biased region:Poly-Arg	5	2.84	239845, 11504, 17388, 23794, 11790	0.0544	3.494
GOTERM_CC_DIRECT	GO:0043234~protein complex	10	5.68	17192, 17313, 18211, 12558, 20969, 18759, 15519, 384783, 12394, 20466	0.0572	2.033
UP_KEYWORDS	Lipid-binding	5	2.84	18667, 76742, 214459, 269224, 16592	0.0591	3.406
UP_SEQ_FEATURE	lipid moiety-binding region:S-palmitoyl cysteine	5	2.84	14432, 11552, 70310, 11539, 13385	0.0603	3.377
GOTERM_MF_DIRECT	GO:0019901~protein kinase binding	8	4.55	12703, 16890, 12558, 15519, 384783, 73047, 13385, 75580	0.0627	2.265
UP_KEYWORDS	Growth regulation	3	1.70	12703, 14432, 207683	0.0628	7.320
GOTERM_BP_DIRECT	GO:0007157~heterophilic cell-cell adhesion via plasma membrane cell adhesion molecules	3	1.70	16402, 12558, 224024	0.0637	7.244
UP_KEYWORDS	Lipoprotein	11	6.25	14432, 18667, 11552, 70310, 320150, 11539, 19340, 13385, 244058, 19338, 19416	0.0653	1.893
UP_KEYWORDS	Cell projection	10	5.68	27418, 18752, 14432, 241116, 140486, 72555, 20392, 13385, 12794, 432611	0.0658	1.979
INTERPRO	IPR013032:EGF-like, conserved site	5	2.84	17183, 73182, 12223, 109685, 224024	0.0667	3.267
GOTERM_MF_DIRECT	GO:0030165~PDZ domain binding	4	2.27	72555, 13385, 14362, 53310	0.0678	4.237
GOTERM_BP_DIRECT	GO:0048705~skeletal system morphogenesis	3	1.70	12842, 14260, 19338	0.0681	6.976
UP_KEYWORDS	SH3 domain	5	2.84	214459, 83813, 17974, 13385, 53310	0.0692	3.226
GOTERM_BP_DIRECT	GO:0034613~cellular protein localization	3	1.70	83409, 18759, 20466	0.0703	6.849
GOTERM_CC_DIRECT	GO:0005911~cell-cell junction	5	2.84	18752, 50918, 16402, 12558, 53310	0.0722	3.176
GOTERM_MF_DIRECT	GO:0004860~protein kinase inhibitor activity	3	1.70	12703, 18767, 73047	0.0776	6.466
UP_KEYWORDS	Acetylation	31	17.61	80288, 67684, 75956, 105663, 18767, 12350, 67959, 269224, 12394, 19340, 319554, 243168, 77254, 230793, 56376, 16592, 13807, 18759, 12349, 71452, 20466, 56812, 66089, 27418, 71994, 21991, 17974, 56390, 17355, 15519, 22628	0.0802	1.331
GOTERM_CC_DIRECT	GO:0005794~Golgi apparatus	15	8.52	218271, 12842, 68166, 18739, 228368, 20452, 19338, 98733, 16402, 269224, 11766, 320150, 20392, 252903, 98170	0.0804	1.609
INTERPRO	IPR001452:Src homology-3 domain	5	2.84	214459, 83813, 17974, 13385, 53310	0.0824	3.036
GOTERM_CC_DIRECT	GO:0005923~bicellular tight junction	4	2.27	54420, 12737, 18759, 53310	0.0826	3.898
GOTERM_BP_DIRECT	GO:0001822~kidney development	4	2.27	11504, 14260, 12349, 21410	0.0841	3.864
KEGG_PATHWAY	mmu04923:Regulation of lipolysis in adipocytes	3	1.70	16890, 11539, 384783	0.0851	6.064
UP_KEYWORDS	EGF-like domain	5	2.84	17183, 73182, 12223, 109685, 224024	0.0855	2.996
KEGG_PATHWAY	mmu05206:MicroRNAs in cancer	6	3.41	18752, 12703, 140486, 16402, 384783, 13435	0.0863	2.505
UP_KEYWORDS	Metal-binding	33	18.75	218271, 17313, 17347, 381924, 20437, 105663, 16399, 13998, 18667, 12350, 93691, 16402, 17388, 23794, 13089, 13435, 319554, 56525, 12842, 56376, 18739, 16882, 13807, 18759, 12349, 213484, 66089, 18752, 140792, 208228, 11504, 12558, 75580	0.0870	1.304
UP_SEQ_FEATURE	transmembrane region	44	25.00	218271, 50918, 60504, 11552, 70310, 381924, 75571, 228368, 11539, 13642, 20452, 224024, 16399, 14764, 241116, 217219, 382551, 16402, 72055, 207683, 17388, 320150, 12723, 98170, 14615, 77254, 54420, 380712, 12737, 12223, 232664, 54445, 12794, 15937, 73182, 140792, 18211, 239845, 18214, 170813, 12558, 20969, 20392, 14362	0.0877	1.234
GOTERM_BP_DIRECT	GO:0016192~vesicle-mediated transport	5	2.84	214459, 68166, 11766, 17974, 252903	0.0890	2.948
GOTERM_MF_DIRECT	GO:0008022~protein C-terminus binding	5	2.84	73569, 20437, 20969, 13385, 53310	0.0896	2.939
UP_KEYWORDS	Ubl conjugation	17	9.66	140486, 67684, 15957, 233863, 20466, 18752, 18211, 94232, 18667, 67959, 140917, 21991, 20392, 15519, 13435, 75580, 14362	0.0925	1.516
GOTERM_CC_DIRECT	GO:0005811~lipid particle	3	1.70	243168, 16890, 18739	0.0935	5.803
GOTERM_BP_DIRECT	GO:0071230~cellular response to amino acid stimulus	4	2.27	12703, 12842, 83409, 13435	0.0949	3.666
KEGG_PATHWAY	mmu05205:Proteoglycans in cancer	5	2.84	18752, 12842, 16402, 20969, 14362	0.0951	2.838
GOTERM_CC_DIRECT	GO:0031234~extrinsic component of cytoplasmic side of plasma membrane	3	1.70	54721, 83813, 13385	0.0983	5.633
GOTERM_BP_DIRECT	GO:0040008~regulation of growth	3	1.70	12703, 14432, 207683	0.1035	5.460
KEGG_PATHWAY	mmu05160:Hepatitis C	4	2.27	54721, 15957, 54420, 12737	0.1102	3.389
UP_KEYWORDS	Endosome	7	3.98	18211, 83409, 76742, 382551, 11766, 18759, 54445	0.1121	2.121
GOTERM_MF_DIRECT	GO:0008565~protein transporter activity	3	1.70	11766, 252903, 54445	0.1125	5.191
UP_KEYWORDS	Cleavage on pair of basic residues	5	2.84	16402, 11504, 17388, 12558, 23794	0.1132	2.706

Supplemental File 3. Continuation.

Category	Term	Count	%	Genes	PValue	Fold Enrichment
UP_KEYWORDS	Lysosome	5	2.84	110173, 83409, 382551, 109685, 54445	0.1142	2.695
GOTERM_BP_DIRECT	GO:0007411~axon guidance	4	2.27	18211, 14432, 93691, 13642	0.1148	3.371
KEGG_PATHWAY	mmu04530:Tight junction	4	2.27	18752, 54420, 12737, 18759	0.1156	3.316
UP_KEYWORDS	Disulfide bond	30	17.05	17313, 60504, 11552, 381924, 11790, 11539, 13642, 20452, 224024, 16399, 14764, 16402, 382551, 17388, 207683, 23794, 244058, 12842, 16592, 12737, 12223, 17183, 140792, 73182, 18211, 98733, 18214, 11504, 109685, 14362	0.1161	1.289
INTERPRO	IPR001478:PDZ domain	4	2.27	56376, 76742, 13385, 53310	0.1168	3.343
GOTERM_MF_DIRECT	GO:0005102~receptor binding	7	3.98	54721, 83813, 13642, 70827, 13385, 244058, 14362	0.1174	2.087
GOTERM_CC_DIRECT	GO:0009986~cell surface	9	5.11	17826, 18211, 227290, 16402, 20969, 15519, 244058, 16399, 14362	0.1183	1.827
UP_SEQ_FEATURE	domain:Protein kinase	8	4.55	18752, 17347, 18211, 18647, 83813, 18214, 269224, 18759	0.1203	1.926
KEGG_PATHWAY	mmu05412:Arrhythmogenic right ventricular cardiomyopathy (ARVC)	3	1.70	16402, 12558, 16399	0.1230	4.869
GOTERM_MF_DIRECT	GO:0051082~unfolded protein binding	3	1.70	227290, 15519, 56812	0.1231	4.914
GOTERM_CC_DIRECT	GO:0015630~microtubule cytoskeleton	4	2.27	227290, 14260, 18759, 67254	0.1253	3.232
GOTERM_CC_DIRECT	GO:0032587~ruffle membrane	3	1.70	67220, 16402, 15519	0.1260	4.848
UP_KEYWORDS	Cell adhesion	7	3.98	16402, 381924, 207683, 12558, 224024, 16399, 12837	0.1266	2.047
GOTERM_BP_DIRECT	GO:0042493~response to drug	6	3.41	12842, 18211, 21410, 15519, 13435, 14362	0.1316	2.222
GOTERM_MF_DIRECT	GO:0001948~glycoprotein binding	3	1.70	20969, 15519, 244058	0.1339	4.666
UP_KEYWORDS	ATP-binding	15	8.52	17347, 54721, 16882, 18759, 11790, 18752, 18211, 83813, 18647, 18214, 269224, 15519, 244059, 233001, 224742	0.1350	1.477
GOTERM_MF_DIRECT	GO:0003779~actin binding	6	3.41	56376, 68166, 14260, 71994, 68524, 22628	0.1392	2.181
UP_KEYWORDS	Postsynaptic cell membrane	4	2.27	239845, 56376, 13385, 12794	0.1425	3.050
INTERPRO	IPR008979:Galactose-binding domain-like	3	1.70	27418, 110173, 18214	0.1427	4.490
UP_SEQ_FEATURE	compositionally biased region:Pro-rich	10	5.68	230793, 73182, 80288, 83813, 14260, 75956, 11790, 71452, 114601, 224024	0.1482	1.654
SMART	SM00228:PDZ	4	2.27	56376, 76742, 13385, 53310	0.1499	2.957
GOTERM_MF_DIRECT	GO:0000166~nucleotide binding	21	11.93	17347, 54721, 140486, 30946, 16882, 11790, 18759, 19338, 18752, 18211, 67959, 18647, 83813, 18214, 269224, 15519, 244059, 224742, 19340, 233001, 19416	0.1499	1.333
GOTERM_BP_DIRECT	GO:0032869~cellular response to insulin stimulus	3	1.70	18759, 384783, 22628	0.1537	4.281
UP_KEYWORDS	Nucleotide-binding	18	10.23	17347, 54721, 16882, 18759, 11790, 19338, 18752, 18211, 83813, 18647, 18214, 269224, 15519, 244059, 224742, 19340, 233001, 19416	0.1544	1.377
UP_SEQ_FEATURE	compositionally biased region:Poly-Pro	6	3.41	76022, 217219, 70310, 68524, 17355, 14362	0.1552	2.102
GOTERM_BP_DIRECT	GO:0007268~chemical synaptic transmission	4	2.27	18752, 14764, 14615, 53310	0.1556	2.920
GOTERM_BP_DIRECT	GO:0060070~canonical Wnt signaling pathway	3	1.70	80288, 20969, 14362	0.1565	4.233
UP_SEQ_FEATURE	domain:SH3	4	2.27	214459, 83813, 13385, 53310	0.1568	2.913
GOTERM_BP_DIRECT	GO:0007568~aging	4	2.27	17192, 18211, 13435, 20466	0.1570	2.903
KEGG_PATHWAY	mmu04514:Cell adhesion molecules (CAMs)	4	2.27	54420, 12558, 12737, 20969	0.1606	2.845
GOTERM_MF_DIRECT	GO:0035091~phosphatidylinositol binding	3	1.70	76742, 18739, 269224	0.1619	4.141
GOTERM_BP_DIRECT	GO:0030154~cell differentiation	10	5.68	17313, 54721, 195434, 18211, 14432, 83813, 75571, 20437, 11790, 13642	0.1654	1.610
KEGG_PATHWAY	mmu04012:ErbB signaling pathway	3	1.70	18752, 17974, 12223	0.1702	3.973
GOTERM_MF_DIRECT	GO:0019900~kinase binding	3	1.70	18211, 13385, 53310	0.1705	4.006
UP_KEYWORDS	Translation regulation	3	1.70	17347, 140486, 17974	0.1722	3.986
GOTERM_CC_DIRECT	GO:0005604~basement membrane	3	1.70	11504, 12394, 12837	0.1745	3.949
GOTERM_MF_DIRECT	GO:0008289~lipid binding	5	2.84	18667, 76742, 214459, 269224, 16592	0.1752	2.275
GOTERM_BP_DIRECT	GO:0001503~ossification	3	1.70	17313, 18214, 12394	0.1760	3.924
UP_SEQ_FEATURE	disulfide bond	26	14.77	17313, 60504, 11552, 381924, 11539, 11790, 13642, 20452, 224024, 16399, 14764, 16402, 382551, 17388, 207683, 23794, 16592, 12223, 17183, 140792, 73182, 18211, 18214, 11504, 109685, 14362	0.1765	1.252
KEGG_PATHWAY	mmu04151:PI3K-Akt signaling pathway	6	3.41	12842, 16402, 21770, 15519, 22628, 16399	0.1801	1.970
UP_KEYWORDS	SH2 domain	3	1.70	54721, 12703, 17974	0.1828	3.834
UP_KEYWORDS	Glycoprotein	34	19.32	218271, 60504, 381924, 11539, 13642, 20452, 224024, 16399, 14764, 16402, 217219, 207683, 17388, 72555, 23794, 98170, 244058, 110173, 12842, 12223, 54445, 15937, 17183, 73182, 140792, 239845, 18211, 18214, 11504, 12558, 20969, 20392, 109685, 14362	0.1885	1.196
GOTERM_CC_DIRECT	GO:0043209~myelin sheath	4	2.27	13807, 12349, 15519, 22628	0.1885	2.660
UP_KEYWORDS	Developmental protein	11	6.25	17313, 195434, 18211, 14432, 75571, 20437, 18759, 13642, 384452, 210719, 14362	0.1886	1.513
GOTERM_CC_DIRECT	GO:0070062~extracellular exosome	26	14.77	17313, 50918, 276919, 16399, 12837, 207683, 406220, 244059, 98170, 244058, 19340, 54721, 16592, 13807, 18759, 12349, 19338, 140792, 83409, 214459, 18214, 12558, 21991, 20969, 22628, 15519	0.1890	1.241
GOTERM_BP_DIRECT	GO:0015031~protein transport	8	4.55	12842, 76742, 68166, 18739, 11766, 252903, 19340, 19338	0.1892	1.697
UP_KEYWORDS	Magnesium	7	3.98	319554, 17347, 381924, 16882, 13807, 105663, 213484	0.1906	1.803
GOTERM_BP_DIRECT	GO:0007275~multicellular organism development	12	6.82	17313, 195434, 18211, 14432, 75571, 20437, 21991, 18759, 13642, 384452, 210719, 14362	0.1915	1.464
GOTERM_BP_DIRECT	GO:0007267~cell-cell signaling	3	1.70	11552, 14362, 14615	0.1960	3.657
UP_KEYWORDS	Differentiation	8	4.55	17313, 195434, 18211, 14432, 75571, 20437, 11790, 13642	0.2040	1.662
UP_KEYWORDS	Repressor	7	3.98	76022, 67959, 12394, 13435, 20466, 75580, 353282	0.2054	1.759
GOTERM_CC_DIRECT	GO:0000139~Golgi membrane	6	3.41	228368, 11766, 320150, 18759, 98170, 19338	0.2059	1.901
GOTERM_CC_DIRECT	GO:0005730~nucleolus	10	5.68	17192, 53414, 17347, 195434, 80288, 30946, 276919, 233863, 20466, 244059	0.2101	1.516
KEGG_PATHWAY	mmu04972:Pancreatic secretion	3	1.70	18752, 12349, 19340	0.2105	3.457
GOTERM_BP_DIRECT	GO:0035556~intracellular signal transduction	6	3.41	18752, 17347, 142688, 54721, 12703, 18759	0.2106	1.884
GOTERM_BP_DIRECT	GO:0001701~in utero embryonic development	5	2.84	17192, 53414, 11790, 12394, 20466	0.2123	2.100
GOTERM_BP_DIRECT	GO:0030335~positive regulation of cell migration	4	2.27	50918, 12842, 16402, 384783	0.2170	2.474
INTERPRO	IPR000980:SH2 domain	3	1.70	54721, 12703, 17974	0.2171	3.417
UP_KEYWORDS	Nucleus	39	22.16	17347, 80288, 67684, 75956, 20437, 30946, 11790, 21410, 233863, 17192, 195434, 12703, 18667, 94232, 76022, 67959, 93691, 18647, 269224, 140917, 13435, 12394, 244059, 224742, 19416, 353282, 140486, 73569, 14260, 16882, 18759, 384452, 210719, 20466, 53414, 67220, 17355, 75580, 73047	0.2218	1.154
KEGG_PATHWAY	mmu04066:HIF-1 signaling pathway	3	1.70	18752, 17347, 13807	0.2231	3.324
GOTERM_CC_DIRECT	GO:0045177~apical part of cell	3	1.70	53414, 12349, 18759	0.2254	3.331
UP_SEQ_FEATURE	compositionally biased region:Poly-Glu	6	3.41	76022, 76156, 11790, 71452, 114601, 17355	0.2284	1.827

Supplemental File 3. Continuation.

Category	Term	Count	%	Genes	PValue	Fold Enrichment
GOTERM_CC_DIRECT	GO:0005783~endoplasmic reticulum	14	7.95	17313, 18667, 12842, 94232, 54420, 16402, 18739, 17974, 11539, 54445, 98170, 13385, 244058, 12794	0.2304	1.351
GOTERM_MF_DIRECT	GO:0046872~metal ion binding	32	18.18	218271, 17347, 381924, 20437, 105663, 13998, 16399, 18667, 12350, 93691, 16402, 17388, 23794, 230761, 13435, 319554, 56525, 12842, 56376, 18739, 13807, 16882, 18759, 12349, 213484, 66089, 18752, 140792, 208228, 11504, 12558, 75580	0.2307	1.172
UP_KEYWORDS	Autophagy	3	1.70	94232, 214459, 19338	0.2314	3.273
GOTERM_CC_DIRECT	GO:0005913~cell-cell adherens junction	5	2.84	208177, 56376, 67959, 71994, 12558	0.2323	2.020
GOTERM_MF_DIRECT	GO:0003677~DNA binding	19	10.80	230793, 67684, 30946, 16882, 21410, 319758, 384452, 210719, 233863, 20466, 17192, 18667, 76022, 67959, 93691, 13435, 244059, 12394, 75580	0.2342	1.264
GOTERM_MF_DIRECT	GO:0005524~ATP binding	16	9.09	17347, 54721, 16882, 18759, 11790, 18752, 18211, 83813, 18647, 18214, 269224, 15519, 244059, 12394, 233001, 224742	0.2359	1.304
KEGG_PATHWAY	mmu04114:Oocyte meiosis	3	1.70	18667, 21770, 22628	0.2421	3.142
UP_KEYWORDS	Tumor suppressor	3	1.70	67220, 320150, 18759	0.2450	3.145
GOTERM_BP_DIRECT	GO:0006417~regulation of translation	3	1.70	17347, 140486, 17974	0.2483	3.113
GOTERM_CC_DIRECT	GO:0005768~endosome	7	3.98	18211, 83409, 76742, 382551, 11766, 18759, 54445	0.2500	1.643
KEGG_PATHWAY	mmu05200:Pathways in cancer	6	3.41	18752, 18211, 15519, 12394, 16399, 14362	0.2511	1.741
GOTERM_BP_DIRECT	GO:0002230~positive regulation of defense response to virus by host	3	1.70	228368, 76156, 20466	0.2512	3.088
GOTERM_CC_DIRECT	GO:0016324~apical plasma membrane	5	2.84	18214, 12558, 12737, 18759, 15519	0.2524	1.946
UP_KEYWORDS	Extracellular matrix	4	2.27	12842, 11504, 23794, 12837	0.2528	2.284
GOTERM_CC_DIRECT	GO:0005769~early endosome	4	2.27	18211, 76742, 382551, 20437	0.2565	2.260
UP_KEYWORDS	Protein transport	7	3.98	76742, 68166, 18739, 11766, 252903, 19340, 19338	0.2570	1.628
SMART	SM00326:SH3	4	2.27	214459, 17974, 13385, 53310	0.2571	2.252
GOTERM_CC_DIRECT	GO:0005634~nucleus	52	29.55	17347, 80288, 75956, 20437, 233863, 70827, 16890, 94232, 93691, 18647, 269224, 140917, 224742, 19416, 54721, 384452, 56812, 66089, 17355, 75580, 67684, 30946, 11790, 21410, 276919, 18767, 17192, 195434, 12703, 18667, 76022, 67959, 244059, 12394, 13435, 353282, 140486, 73569, 14260, 16882, 18759, 319758, 210719, 105203, 20466, 15937, 53414, 18752, 67220, 21991, 15519, 73047	0.2574	1.103
GOTERM_CC_DIRECT	GO:0005764~lysosome	5	2.84	110173, 83409, 382551, 109685, 54445	0.2575	1.929
KEGG_PATHWAY	mmu04510:Focal adhesion	4	2.27	18752, 12842, 16402, 16399	0.2601	2.227
UP_SEQ_FEATURE	nucleotide phosphate-binding region:ATP	11	6.25	18752, 17347, 54721, 18211, 18647, 83813, 18214, 269224, 18759, 11790, 233001	0.2683	1.381
INTERPRO	IPR000742:Epidermal growth factor-like domain	4	2.27	17183, 73182, 12223, 224024	0.2734	2.172
INTERPRO	IPR009057:Homeodomain-like	5	2.84	76022, 21410, 384452, 210719, 244059	0.2770	1.865
KEGG_PATHWAY	mmu04670:Leukocyte transendothelial migration	3	1.70	18752, 54420, 12737	0.2770	2.857
GOTERM_BP_DIRECT	GO:0006886~intracellular protein transport	4	2.27	76742, 11766, 252903, 54445	0.2782	2.165
KEGG_PATHWAY	mmu04142:Lysosome	3	1.70	110173, 11766, 252903	0.2802	2.833
GOTERM_BP_DIRECT	GO:0018105~peptidyl-serine phosphorylation	3	1.70	18752, 17347, 18759	0.2837	2.832
KEGG_PATHWAY	mmu04071:Sphingolipid signaling pathway	3	1.70	18752, 21770, 11539	0.2866	2.788
UP_KEYWORDS	Actin-binding	4	2.27	68166, 14260, 71994, 68524	0.2873	2.130
GOTERM_MF_DIRECT	GO:0044822~poly(A) RNA binding	12	6.82	53414, 241116, 67959, 67684, 140486, 76917, 75956, 30946, 15519, 22628, 244059, 224742	0.2889	1.325
UP_KEYWORDS	Zinc	19	10.80	56525, 17347, 56376, 20437, 16882, 12349, 18759, 66089, 13998, 18752, 18667, 12350, 208228, 93691, 11504, 17388, 23794, 13435, 75580	0.2920	1.215
GOTERM_CC_DIRECT	GO:0048471~perinuclear region of cytoplasm	8	4.55	18752, 53414, 94232, 140486, 98733, 68166, 15519, 19416	0.2938	1.476
UP_KEYWORDS	Lyase	3	1.70	12350, 13807, 12349	0.2944	2.758
KEGG_PATHWAY	mmu04152:AMPK signaling pathway	3	1.70	16890, 21770, 384783	0.2961	2.722
GOTERM_MF_DIRECT	GO:0005198~structural molecule activity	4	2.27	54420, 406220, 12737, 13385	0.2980	2.082
UP_KEYWORDS	DNA-binding	15	8.52	230793, 67684, 30946, 21410, 384452, 210719, 233863, 17192, 18667, 67959, 93691, 244059, 12394, 13435, 75580	0.3011	1.255
GOTERM_BP_DIRECT	GO:0008360~regulation of cell shape	3	1.70	27418, 67220, 68524	0.3013	2.710
GOTERM_BP_DIRECT	GO:0045893~positive regulation of transcription, DNA-templated	7	3.98	12842, 18667, 93691, 21410, 17355, 12394, 14362	0.3049	1.526
GOTERM_CC_DIRECT	GO:0005938~cell cortex	3	1.70	27418, 214459, 68166	0.3088	2.660
KEGG_PATHWAY	mmu04611:Platelet activation	3	1.70	12842, 18759, 16399	0.3088	2.639
GOTERM_BP_DIRECT	GO:0006914~autophagy	3	1.70	94232, 214459, 19338	0.3100	2.653
GOTERM_MF_DIRECT	GO:0016829~lyase activity	3	1.70	12350, 13807, 12349	0.3112	2.652
UP_SEQ_FEATURE	glycosylation site:N-linked (GlcNAc...)	33	18.75	218271, 60504, 381924, 11539, 13642, 20452, 224024, 16399, 14764, 16402, 217219, 207683, 17388, 23794, 98170, 244058, 110173, 12842, 12223, 54445, 15937, 17183, 73182, 140792, 239845, 18211, 18214, 11504, 12558, 20969, 20392, 109685, 14362	0.3168	1.120
GOTERM_MF_DIRECT	GO:0003729~mRNA binding	3	1.70	67684, 140486, 15519	0.3188	2.596
GOTERM_CC_DIRECT	GO:0030424~axon	5	2.84	18211, 14432, 18667, 140486, 12349	0.3253	1.725
GOTERM_MF_DIRECT	GO:0032403~protein complex binding	5	2.84	12558, 17974, 21410, 20466, 13385	0.3285	1.716
INTERPRO	IPR001849:Pleckstrin homology domain	4	2.27	208177, 67220, 384783, 13998	0.3297	1.965
KEGG_PATHWAY	mmu04310:Wnt signaling pathway	3	1.70	18752, 20437, 14362	0.3403	2.452
UP_KEYWORDS	Transferase	15	8.52	218271, 17347, 54721, 18759, 11790, 20452, 18752, 18211, 83813, 18647, 18214, 269224, 320150, 13435, 73047	0.3418	1.217
GOTERM_CC_DIRECT	GO:0005667~transcription factor complex	4	2.27	30946, 21410, 210719, 20466	0.3445	1.913
GOTERM_MF_DIRECT	GO:0008201~heparin binding	3	1.70	227290, 11504, 23794	0.3455	2.441
KEGG_PATHWAY	mmu04630:Jak-STAT signaling pathway	3	1.70	54721, 12703, 60504	0.3528	2.384
GOTERM_BP_DIRECT	GO:0043524~negative regulation of neuron apoptotic process	3	1.70	18752, 18211, 18759	0.3617	2.354
GOTERM_CC_DIRECT	GO:0005802~trans-Golgi network	3	1.70	214459, 11766, 17974	0.3653	2.336
GOTERM_MF_DIRECT	GO:0008270~zinc ion binding	11	6.25	18752, 18667, 56376, 12350, 11504, 20437, 17388, 16882, 23794, 320150, 12349	0.3700	1.257
UP_SEQ_FEATURE	compositionally biased region:Asp/Glu-rich (acidic)	3	1.70	11552, 71994, 233863	0.3880	2.225
INTERPRO	IPR011993:Pleckstrin homology-like domain	5	2.84	208177, 67220, 54721, 384783, 13998	0.3885	1.574
GOTERM_BP_DIRECT	GO:0060271~cilium morphogenesis	3	1.70	214459, 384452, 432611	0.3901	2.216

Supplemental File 3. Continuation.

Category	Term	Count	%	Genes	PValue	Fold Enrichment
GOTERM_MF_DIRECT	GO:0098641~cadherin binding involved in cell-cell adhesion	4	2.27	208177, 56376, 67959, 71994	0.3928	1.761
SMART	SM00233:PH	4	2.27	208177, 67220, 384783, 13998	0.3936	1.753
UP_SEQ_FEATURE	compositionally biased region:Poly-Lys	3	1.70	73884, 76022, 30946	0.3975	2.185
GOTERM_CC_DIRECT	GO:0043025~neuronal cell body	6	3.41	18211, 71994, 13807, 11539, 15519, 53310	0.4020	1.435
UP_KEYWORDS	Transcription	16	9.09	80288, 73569, 30946, 21410, 384452, 233863, 20466, 17192, 18667, 76022, 67959, 93691, 244059, 12394, 13435, 75580	0.4040	1.155
UP_KEYWORDS	Spermatogenesis	3	1.70	195434, 75571, 20437	0.4067	2.142
UP_SEQ_FEATURE	compositionally biased region:Poly-Ala	4	2.27	73884, 30946, 23794, 244058	0.4073	1.721
UP_KEYWORDS	Isopeptide bond	9	5.11	18667, 76022, 67959, 67684, 140486, 21991, 233863, 224742, 75580	0.4103	1.267
GOTERM_MF_DIRECT	GO:0016740~transferase activity	14	7.95	218271, 17347, 54721, 18759, 11790, 20452, 18752, 18211, 83813, 18647, 18214, 269224, 320150, 13435	0.4115	1.168
GOTERM_MF_DIRECT	GO:0019904~protein domain specific binding	4	2.27	18759, 384783, 22628, 53310	0.4145	1.700
UP_SEQ_FEATURE	signal peptide	28	15.91	17313, 60504, 381924, 13642, 224024, 16399, 12837, 16402, 382551, 217219, 17388, 207683, 23794, 98170, 244058, 243168, 110173, 12842, 12223, 17183, 73182, 18211, 18214, 11504, 12558, 20969, 109685, 14362	0.4193	1.083
GOTERM_BP_DIRECT	GO:0006351~transcription, DNA-templated	17	9.66	80288, 73569, 30946, 21410, 384452, 233863, 20466, 17192, 18667, 76022, 67959, 93691, 17355, 244059, 12394, 13435, 75580	0.4217	1.132
KEGG_PATHWAY	mmu04141:Protein processing in endoplasmic reticulum	3	1.70	94232, 15519, 56812	0.4229	2.058
UP_KEYWORDS	Coiled coil	25	14.20	208177, 73884, 67684, 15957, 212706, 14260, 75956, 30946, 232664, 114601, 70827, 20466, 17183, 17192, 195434, 140792, 239845, 66611, 241116, 214459, 406220, 72055, 15519, 75580, 224742	0.4254	1.091
GOTERM_BP_DIRECT	GO:0001934~positive regulation of protein phosphorylation	3	1.70	18211, 244058, 14362	0.4285	2.047
KEGG_PATHWAY	mmu04022:cGMP-PKG signaling pathway	3	1.70	11552, 11539, 384783	0.4317	2.021
GOTERM_MF_DIRECT	GO:0005516~calmodulin binding	3	1.70	17347, 14432, 71994	0.4339	2.025
GOTERM_BP_DIRECT	GO:0098609~cell-cell adhesion	3	1.70	56376, 67959, 71994	0.4419	1.993
GOTERM_CC_DIRECT	GO:0005578~proteinaceous extracellular matrix	4	2.27	12842, 11504, 23794, 12837	0.4470	1.616
GOTERM_CC_DIRECT	GO:0009897~external side of plasma membrane	4	2.27	16402, 20969, 432611, 16399	0.4491	1.611
UP_SEQ_FEATURE	mutagenesis site	8	4.55	12703, 83813, 11504, 21991, 18759, 12394, 13435, 20466	0.4501	1.253
UP_SEQ_FEATURE	binding site:Substrate	4	2.27	319554, 243168, 13807, 21991	0.4572	1.591
UP_KEYWORDS	Polymorphism	3	1.70	17183, 60504, 21410	0.4606	1.926
UP_KEYWORDS	Transcription regulation	15	8.52	80288, 73569, 30946, 21410, 384452, 20466, 17192, 18667, 76022, 67959, 93691, 244059, 12394, 13435, 75580	0.4643	1.119
GOTERM_BP_DIRECT	GO:0043066~negative regulation of apoptotic process	6	3.41	18667, 12223, 11539, 21410, 20466, 15937	0.4651	1.331
GOTERM_MF_DIRECT	GO:0042803~protein homodimerization activity	8	4.55	18211, 20437, 13807, 12723, 21410, 15519, 75580, 14362	0.4674	1.232
GOTERM_CC_DIRECT	GO:0005622~intracellular	14	7.95	56525, 18752, 142688, 14764, 208228, 11552, 76742, 18739, 269224, 13807, 18759, 15519, 19340, 19338	0.4768	1.118
UP_KEYWORDS	Immunoglobulin domain	5	2.84	18211, 98733, 382551, 207683, 11790	0.4786	1.395
GOTERM_BP_DIRECT	GO:0045892~negative regulation of transcription, DNA-templated	6	3.41	76022, 12394, 20466, 75580, 14362, 19416	0.4850	1.301
INTERPRO	IPR003961:Fibronectin, type III	3	1.70	60504, 98733, 11790	0.4861	1.830
GOTERM_BP_DIRECT	GO:0005975~carbohydrate metabolic process	3	1.70	218271, 110173, 109685	0.4864	1.829
GOTERM_MF_DIRECT	GO:0000287~magnesium ion binding	3	1.70	13807, 105663, 213484	0.4875	1.825
GOTERM_CC_DIRECT	GO:0005856~cytoskeleton	10	5.68	54721, 214459, 68166, 14260, 140917, 406220, 68524, 20392, 432611, 13998	0.5018	1.147
UP_SEQ_FEATURE	active site:Proton donor	3	1.70	110173, 13807, 109685	0.5040	1.769
UP_KEYWORDS	Signal	35	19.89	17313, 60504, 381924, 13642, 20452, 224024, 16399, 12837, 18647, 382551, 16402, 217219, 17388, 207683, 72555, 23794, 13089, 98170, 244058, 243168, 100043125, 110173, 12842, 73833, 12223, 17183, 73182, 18211, 18214, 11504, 12558, 20969, 20392, 109685, 14362	0.5041	1.034
KEGG_PATHWAY	mmu05016:Huntington's disease	3	1.70	20466, 13385, 432611	0.5082	1.746
UP_SEQ_FEATURE	domain:PH	3	1.70	208177, 67220, 384783	0.5117	1.744
GOTERM_BP_DIRECT	GO:0000122~negative regulation of transcription from RNA polymerase II promoter	7	3.98	17192, 21410, 210719, 13435, 20466, 18767, 75580	0.5190	1.206
GOTERM_CC_DIRECT	GO:0043231~intracellular membrane-bounded organelle	7	3.98	53414, 320150, 13089, 12394, 244059, 353282, 12837	0.5322	1.190
GOTERM_BP_DIRECT	GO:0045944~positive regulation of transcription from RNA polymerase II promoter	9	5.11	18667, 80288, 21770, 17974, 21410, 210719, 12394, 20466, 244058	0.5326	1.136
GOTERM_MF_DIRECT	GO:0000977~RNA polymerase II regulatory region sequence-specific DNA binding	3	1.70	21410, 12394, 75580	0.5355	1.668
GOTERM_BP_DIRECT	GO:0006355~regulation of transcription, DNA-templated	19	10.80	56525, 80288, 73569, 30946, 21410, 319758, 384452, 210719, 20466, 17192, 18667, 76022, 67959, 93691, 244059, 12394, 13435, 75580, 353282	0.5404	1.047
GOTERM_CC_DIRECT	GO:0005654~nucleoplasm	16	9.09	17347, 80288, 67684, 76742, 21410, 105203, 20466, 17192, 67959, 76156, 15519, 244059, 12394, 13435, 224742, 353282	0.5462	1.056
UP_KEYWORDS	Neurogenesis	3	1.70	18211, 14432, 13642	0.5482	1.630
KEGG_PATHWAY	mmu01130:Biosynthesis of antibiotics	3	1.70	319554, 13807, 21991	0.5503	1.615
KEGG_PATHWAY	mmu04015:Rap1 signaling pathway	3	1.70	18752, 18759, 16399	0.5503	1.615
INTERPRO	IPR027417:P-loop containing nucleoside triphosphate hydrolase	8	4.55	244059, 233001, 19340, 13385, 224742, 19338, 19416, 53310	0.5570	1.133
GOTERM_MF_DIRECT	GO:0000978~RNA polymerase II core promoter proximal region sequence-specific DNA binding	4	2.27	17192, 18667, 319758, 210719	0.5574	1.369
GOTERM_BP_DIRECT	GO:0001525~angiogenesis	3	1.70	11552, 13642, 12837	0.5661	1.576
UP_KEYWORDS	Receptor	20	11.36	60504, 11552, 381924, 258595, 11539, 384783, 224024, 16399, 17826, 140792, 18211, 239845, 14764, 18667, 18214, 16402, 170813, 382551, 207683, 14362	0.5705	1.027
GOTERM_BP_DIRECT	GO:0007399~nervous system development	4	2.27	18211, 14432, 13642, 53310	0.5758	1.332
UP_SEQ_FEATURE	propeptide:Removed in mature form	3	1.70	12223, 244058, 19416	0.5847	1.524
GOTERM_BP_DIRECT	GO:0008285~negative regulation of cell proliferation	4	2.27	18211, 11539, 12394, 56812	0.5884	1.308
GOTERM_MF_DIRECT	GO:0046982~protein heterodimerization activity	5	2.84	381924, 13807, 11539, 21410, 14362	0.6002	1.195
GOTERM_MF_DIRECT	GO:0005525~GTP binding	4	2.27	15519, 19340, 19338, 19416	0.6014	1.283
GOTERM_MF_DIRECT	GO:0004871~signal transducer activity	6	3.41	14764, 239845, 320150, 11539, 384783, 14362	0.6048	1.138
GOTERM_BP_DIRECT	GO:0045087~innate immune response	4	2.27	54721, 15957, 83813, 54445	0.6161	1.256
GOTERM_BP_DIRECT	GO:0010629~negative regulation of gene expression	3	1.70	50918, 18667, 353282	0.6225	1.422

Supplemental File 3. Continuation.

Category	Term	Count	%	Genes	PValue	Fold Enrichment
UP_SEQ_FEATURE	compositionally biased region:Ser-rich	4	2.27	208177, 75956, 12558, 232664	0.6248	1.240
GOTERM_BP_DIRECT	GO:0008284~positive regulation of cell proliferation	5	2.84	18667, 18214, 12223, 13642, 384783	0.6248	1.158
GOTERM_BP_DIRECT	GO:0007283~spermatogenesis	4	2.27	195434, 75571, 20437, 13435	0.6279	1.234
UP_KEYWORDS	Signal-anchor	4	2.27	218271, 140792, 20452, 15937	0.6343	1.223
INTERPRO	IPR012677:Nucleotide-binding, alpha-beta plait	3	1.70	67959, 140486, 30946	0.6410	1.374
KEGG_PATHWAY	mmu04010:MAPK signaling pathway	3	1.70	18752, 17347, 18211	0.6423	1.366
GOTERM_BP_DIRECT	GO:0006974~cellular response to DNA damage stimulus	4	2.27	140917, 16882, 244059, 75580	0.6490	1.196
GOTERM_CC_DIRECT	GO:0005789~endoplasmic reticulum membrane	6	3.41	77254, 94232, 18739, 54445, 98170, 12794	0.6516	1.079
UP_SEQ_FEATURE	repeat:LRR 2	3	1.70	18211, 232664, 233001	0.6613	1.324
UP_SEQ_FEATURE	repeat:LRR 1	3	1.70	18211, 232664, 233001	0.6613	1.324
INTERPRO	IPR013083:Zinc finger, RING/FYVE/PHD-type	4	2.27	68166, 20437, 66089, 13998	0.6711	1.157
UP_KEYWORDS	Activator	5	2.84	80288, 93691, 21410, 12394, 224742	0.6825	1.075
UP_KEYWORDS	Cytoskeleton	8	4.55	214459, 68166, 14260, 140917, 68524, 20392, 432611, 13998	0.6870	1.001
UP_SEQ_FEATURE	sequence variant	4	2.27	17183, 60504, 21410, 384452	0.6883	1.127
KEGG_PATHWAY	mmu04144:Endocytosis	3	1.70	18211, 68524, 18759	0.6933	1.243
UP_KEYWORDS	Apoptosis	4	2.27	17347, 67959, 70310, 20437	0.7053	1.098
KEGG_PATHWAY	mmu04080:Neuroactive ligand-receptor interaction	3	1.70	239845, 11552, 11539	0.7065	1.213
GOTERM_BP_DIRECT	GO:0008152~metabolic process	4	2.27	16890, 110173, 21991, 109685	0.7128	1.085
GOTERM_CC_DIRECT	GO:0030529~intracellular ribonucleoprotein complex	3	1.70	67959, 140486, 233863	0.7144	1.197
GOTERM_MF_DIRECT	GO:0003700~transcription factor activity, sequence-specific DNA binding	7	3.98	18667, 93691, 21410, 319758, 17355, 12394, 20466	0.7242	0.974
UP_KEYWORDS	Zinc-finger	11	6.25	56525, 18752, 18667, 93691, 20437, 16882, 18759, 13435, 75580, 66089, 13998	0.7301	0.943
GOTERM_CC_DIRECT	GO:0005694~chromosome	3	1.70	17192, 140917, 75580	0.7510	1.113
INTERPRO	IPR016024:Armadillo-type fold	3	1.70	21770, 11766, 76813	0.7611	1.091
GOTERM_CC_DIRECT	GO:0005887~integral component of plasma membrane	8	4.55	18211, 14764, 11552, 18214, 17388, 12737, 12723, 20392	0.7797	0.907
GOTERM_BP_DIRECT	GO:0016567~protein ubiquitination	3	1.70	142688, 12703, 20437	0.7837	1.041
GOTERM_BP_DIRECT	GO:0006810~transport	13	7.39	140486, 76742, 68166, 18739, 16592, 228368, 19338, 12794, 72055, 11766, 12723, 252903, 19340	0.7879	0.896
UP_KEYWORDS	Immunity	3	1.70	15957, 382551, 54445	0.8002	1.004
GOTERM_MF_DIRECT	GO:0016787~hydrolase activity	11	6.25	319554, 16890, 110173, 11504, 140917, 17388, 23794, 109685, 105663, 244059, 213484	0.8033	0.882
UP_KEYWORDS	Chromosome	3	1.70	17192, 140917, 75580	0.8090	0.984
GOTERM_BP_DIRECT	GO:0002376~immune system process	3	1.70	15957, 382551, 54445	0.8095	0.984
GOTERM_MF_DIRECT	GO:0019899~enzyme binding	3	1.70	18667, 12558, 70827	0.8202	0.960
GOTERM_BP_DIRECT	GO:0006357~regulation of transcription from RNA polymerase II promoter	3	1.70	30946, 319758, 70827	0.8251	0.949
UP_KEYWORDS	RNA-binding	4	2.27	15957, 67959, 140486, 30946	0.8258	0.893
GOTERM_BP_DIRECT	GO:0006915~apoptotic process	4	2.27	17347, 67959, 70310, 20437	0.8329	0.881
GOTERM_CC_DIRECT	GO:0005615~extracellular space	10	5.68	17313, 16890, 12842, 110173, 12223, 13807, 21991, 23794, 12349, 53310	0.8361	0.849
UP_KEYWORDS	Transport	12	6.82	140486, 76742, 68166, 18739, 228368, 16592, 72055, 11766, 12723, 252903, 19340, 19338	0.8421	0.847
GOTERM_BP_DIRECT	GO:0006508~proteolysis	4	2.27	74616, 11504, 17388, 23794	0.8432	0.863
INTERPRO	IPR007110:Immunoglobulin-like domain	6	3.41	100043125, 18211, 98733, 382551, 207683, 11790	0.8434	0.839
GOTERM_CC_DIRECT	GO:0005813~centrosome	3	1.70	98733, 140917, 73047	0.8555	0.881
INTERPRO	IPR013783:Immunoglobulin-like fold	7	3.98	100043125, 60504, 18211, 98733, 382551, 207683, 11790	0.8574	0.820
UP_KEYWORDS	Endoplasmic reticulum	6	3.41	94232, 18739, 17974, 54445, 98170, 12794	0.8661	0.808
UP_KEYWORDS	Hydrolase	10	5.68	16890, 110173, 11504, 140917, 17388, 23794, 109685, 105663, 244059, 213484	0.8677	0.815
UP_SEQ_FEATURE	topological domain:Lumenal	3	1.70	218271, 320150, 20452	0.8692	0.849
GOTERM_BP_DIRECT	GO:0007165~signal transduction	8	4.55	14764, 239845, 76742, 21770, 11539, 384783, 14362, 19416	0.8751	0.800
KEGG_PATHWAY	mmu01100:Metabolic pathways	9	5.11	319554, 218271, 13807, 21991, 13089, 109685, 70827, 13435, 105663	0.8758	0.811
GOTERM_MF_DIRECT	GO:0003723~RNA binding	5	2.84	15957, 67959, 140486, 30946, 20466	0.8806	0.788
UP_KEYWORDS	Secreted	10	5.68	17183, 243168, 17313, 12842, 11504, 12223, 20969, 23794, 109685, 12837	0.8844	0.796
GOTERM_MF_DIRECT	GO:0043565~sequence-specific DNA binding	4	2.27	18667, 21410, 210719, 75580	0.8902	0.776
GOTERM_MF_DIRECT	GO:0003682~chromatin binding	3	1.70	17192, 13435, 20466	0.8939	0.791
GOTERM_MF_DIRECT	GO:0005509~calcium ion binding	4	2.27	17183, 17313, 17388, 12558	0.9254	0.703
INTERPRO	IPR013106:Immunoglobulin V-set	3	1.70	100043125, 382551, 207683	0.9301	0.697
GOTERM_CC_DIRECT	GO:0005576~extracellular region	10	5.68	17183, 243168, 17313, 12842, 11504, 12223, 20969, 23794, 109685, 12837	0.9359	0.728
UP_KEYWORDS	Cell cycle	3	1.70	18647, 20437, 16882	0.9482	0.643
UP_KEYWORDS	Ubl conjugation pathway	3	1.70	142688, 12703, 20437	0.9498	0.638
UP_KEYWORDS	Oxidoreductase	3	1.70	243168, 13089, 22628	0.9523	0.630
GOTERM_BP_DIRECT	GO:0007049~cell cycle	3	1.70	18647, 20437, 16882	0.9574	0.614
GOTERM_CC_DIRECT	GO:0005739~mitochondrion	9	5.11	319554, 16890, 18667, 70310, 16882, 232664, 70827, 15937, 19340	0.9631	0.668
GOTERM_MF_DIRECT	GO:0003676~nucleic acid binding	6	3.41	56525, 93691, 67959, 140486, 230761, 75580	0.9751	0.596
UP_KEYWORDS	Transducer	7	3.98	14764, 239845, 11552, 258595, 11539, 384783, 14362	0.9933	0.522
UP_KEYWORDS	G-protein coupled receptor	6	3.41	14764, 239845, 11552, 258595, 11539, 14362	0.9970	0.462
GOTERM_BP_DIRECT	GO:0007186~G-protein coupled receptor signaling pathway	6	3.41	14764, 239845, 11552, 258595, 11539, 14362	0.9982	0.442
INTERPRO	IPR000276:G protein-coupled receptor, rhodopsin-like	4	2.27	14764, 11552, 258595, 11539	0.9993	0.353
GOTERM_MF_DIRECT	GO:0004930~G-protein coupled receptor activity	5	2.84	14764, 239845, 258595, 11539, 14362	0.9997	0.351
INTERPRO	IPR017452:GPCR, rhodopsin-like, 7TM	4	2.27	14764, 11552, 258595, 11539	0.9999	0.301

Supplemental File 4.

Functional terms not clustered during pathway enrichment analysis of Downregulated genes.

Category	Term	Count	%	Genes	PValue	Fold Enrichment
UP_KEYWORDS	**Lipoprotein**	14	9.33	19342, 106042, 14429, 170638, 67168, 24013, 23912, 14360, 110265, 22421, 13518, 14681, 14701, 110855	0.001	2.971
UP_KEYWORDS	**Prenylation**	6	4.00	19342, 106042, 24013, 23912, 14701, 110855	0.003	6.327
UP_KEYWORDS	**Zinc**	24	16.00	331374, 107823, 100233208, 100041433, 20182, 70081, 433520, 241073, 17764, 106042, 112415, 16876, 22380, 56490, 170753, 74042, 328274, 227059, 67785, 68350, 18105, 433791, 19415, 30791	0.003	1.893
INTERPRO	**IPR007087:Zinc finger, C2H2**	12	8.00	110593, 112415, 170753, 56490, 74042, 328274, 100233208, 100041433, 70081, 433791, 433520, 17764	0.005	2.662
UP_KEYWORDS	**Zinc-finger**	19	12.67	331374, 107823, 100233208, 100041433, 20182, 70081, 241073, 433520, 17764, 112415, 22380, 56490, 170753, 74042, 328274, 67785, 68350, 433791, 19415	0.006	2.010
UP_KEYWORDS	**Glycoprotein**	35	23.33	14254, 14172, 257630, 103963, 73382, 52020, 60505, 18208, 67374, 72043, 13406, 212114, 13838, 20499, 56693, 214523, 67168, 104099, 19128, 74488, 227059, 22421, 63959, 85031, 71844, 108115, 22352, 12164, 74048, 76943, 15247, 14429, 12519, 14365, 268934	0.010	1.519
UP_KEYWORDS	**Metal-binding**	32	21.33	107823, 100233208, 100041433, 20182, 70081, 241073, 17764, 72043, 18570, 106042, 16876, 170638, 170753, 13086, 104099, 67785, 50770, 68350, 14681, 18105, 19415, 331374, 13518, 433520, 112415, 22380, 56490, 74042, 328274, 14360, 433791, 110855	0.010	1.560
GOTERM_BP_DIRECT	**GO:0050770~regulation of axonogenesis**	3	2.00	13838, 22421, 22352	0.015	16.011
GOTERM_BP_DIRECT	**GO:0051216~cartilage development**	4	2.67	12164, 257630, 22421, 72043	0.017	7.290
UP_KEYWORDS	**Myristate**	5	3.33	170638, 14360, 110265, 13518, 14681	0.020	4.812
UP_SEQ_FEATURE	**domain:SH3**	5	3.33	19342, 52906, 14360, 320664, 13518	0.021	4.759
UP_SEQ_FEATURE	**compositionally biased region:Poly-Lys**	5	3.33	15247, 106042, 22380, 52906, 57752	0.021	4.759
UP_KEYWORDS	**Alternative splicing**	40	26.67	107823, 257630, 320664, 52020, 20182, 72776, 67434, 18570, 329251, 74211, 142687, 13838, 20499, 16876, 12695, 235633, 52906, 67785, 63959, 68350, 14681, 71844, 72685, 66609, 108115, 110265, 13518, 106347, 541610, 215351, 56490, 74042, 70461, 622554, 50493, 14360, 12519, 57752, 110855, 268934	0.022	1.386
INTERPRO	**IPR001909:Krueppel-associated box**	7	4.67	112415, 328274, 100233208, 100041433, 70081, 433791, 433520	0.027	3.043
UP_SEQ_FEATURE	**signal peptide**	29	19.33	14254, 14172, 257630, 103963, 73382, 52020, 60505, 18208, 67374, 72043, 13406, 212114, 19342, 13838, 56693, 19128, 74488, 227059, 22421, 85031, 106347, 541610, 12164, 74048, 76943, 228413, 12519, 14365, 268934	0.028	1.467
GOTERM_CC_DIRECT	**GO:0005622~intracellular**	18	12.00	331374, 66399, 100233208, 100041433, 60505, 70081, 433520, 19342, 142687, 112415, 170638, 27263, 328274, 23912, 74488, 433791, 19415, 268934	0.028	1.729
GOTERM_BP_DIRECT	**GO:0007169~transmembrane receptor protein tyrosine kinase signaling pathway**	4	2.67	14254, 13838, 52906, 14360	0.029	5.978
UP_SEQ_FEATURE	**glycosylation site:N-linked (GlcNAc...)**	32	21.33	14254, 14172, 257630, 103963, 73382, 52020, 60505, 18208, 67374, 72043, 13406, 212114, 13838, 20499, 56693, 214523, 67168, 19128, 74488, 227059, 22421, 63959, 85031, 108115, 12164, 74048, 76943, 15247, 14429, 12519, 14365, 268934	0.030	1.419
UP_SEQ_FEATURE	**lipid moiety-binding region:S-geranylgeranyl cysteine**	4	2.67	19342, 23912, 14701, 110855	0.032	5.745
GOTERM_BP_DIRECT	**GO:0046777~protein autophosphorylation**	5	3.33	14254, 13838, 24013, 14360, 22241	0.034	4.083
GOTERM_BP_DIRECT	**GO:0007409~axonogenesis**	4	2.67	18223, 22241, 18208, 13518	0.034	5.586
SMART	**SM00349:KRAB**	7	4.67	112415, 328274, 100233208, 100041433, 70081, 433791, 433520	0.038	2.762
GOTERM_CC_DIRECT	**GO:0055037~recycling endosome**	4	2.67	19342, 78670, 55943, 50770	0.039	5.297
REACTOME_PATHWAY	**R-MMU-202733:R-MMU-202733**	3	2.00	14360, 19128, 67374	0.043	8.912
UP_SEQ_FEATURE	**splice variant**	39	26.00	107823, 257630, 320664, 52020, 20182, 72776, 78748, 67434, 329251, 74211, 142687, 13838, 20499, 16876, 12695, 235633, 52906, 67785, 63959, 68350, 14681, 71844, 72685, 66609, 108115, 13518, 106347, 541610, 215351, 74222, 56490, 74042, 70461, 50493, 14360, 12519, 57752, 110855, 268934	0.044	1.314
UP_KEYWORDS	Phosphoprotein	56	37.33	107823, 320664, 55943, 17135, 69871, 19342, 329251, 74211, 16549, 170753, 18223, 20382, 52906, 20957, 227059, 63959, 19415, 15526, 16572, 108115, 223739, 22352, 22380, 56490, 70461, 233011, 14254, 19265, 66399, 72776, 17764, 13406, 67434, 106042, 13838, 20499, 12695, 24013, 67785, 22241, 50770, 71844, 18105, 72685, 13518, 106347, 215351, 74048, 74042, 228413, 50493, 14360, 57752, 68510, 223453, 14701	0.056	1.217
GOTERM_BP_DIRECT	GO:0006979~response to oxidative stress	4	2.67	331374, 50493, 110265, 17764	0.059	4.494
GOTERM_CC_DIRECT	GO:0031965~nuclear membrane	5	3.33	18570, 106042, 74042, 71844, 68510	0.059	3.398
INTERPRO	IPR020846:Major facilitator superfamily domain	4	2.67	228993, 15247, 108115, 63959	0.061	4.443
UP_KEYWORDS	Nucleotide-binding	17	11.33	16572, 14254, 331374, 74222, 19342, 13838, 76889, 27263, 24013, 23912, 14360, 50770, 22241, 14681, 233011, 110855, 15526	0.061	1.605
UP_KEYWORDS	Signal	36	24.00	14254, 19265, 14172, 257630, 103963, 73382, 52020, 60505, 18208, 628185, 67374, 72043, 13406, 212114, 13838, 56693, 241589, 104099, 19128, 74488, 227059, 22421, 85031, 30791, 106347, 541610, 12164, 74048, 76943, 56490, 93708, 228413, 12519, 14365, 629761, 268934	0.063	1.312
GOTERM_CC_DIRECT	GO:0005768~endosome	8	5.33	18570, 19342, 14254, 13838, 14360, 55943, 50770, 225861	0.063	2.259
UP_KEYWORDS	Kinase	9	6.00	14254, 13838, 76889, 331374, 27263, 24013, 14360, 22241, 233011	0.063	2.107
KEGG_PATHWAY	mmu04014:Ras signaling pathway	5	3.33	14254, 14172, 85031, 14701, 19415	0.064	3.242
REACTOME_PATHWAY	R-MMU-1257604:R-MMU-1257604	3	2.00	14172, 14360, 12519	0.068	6.855
GOTERM_CC_DIRECT	GO:0005884~actin filament	3	2.00	21916, 14360, 14701	0.072	6.777
GOTERM_BP_DIRECT	GO:0007165~signal transduction	14	9.33	14172, 78748, 329251, 170638, 67168, 24013, 22421, 22241, 14365, 14681, 14701, 19415, 268934, 110855	0.073	1.667
GOTERM_BP_DIRECT	GO:0006919~activation of cysteine-type endopeptidase activity involved in apoptotic process	3	2.00	18570, 68350, 223453	0.073	6.691
GOTERM_BP_DIRECT	GO:0007411~axon guidance	4	2.67	16572, 13838, 14360, 18208	0.077	4.012
KEGG_PATHWAY	mmu04390:Hippo signaling pathway	4	2.67	12164, 12695, 22421, 14365	0.077	3.933
UP_KEYWORDS	Cell membrane	30	20.00	14254, 52020, 628185, 241073, 67374, 19342, 13838, 20499, 12695, 259111, 67168, 258713, 50770, 63959, 14681, 30791, 108115, 13518, 106347, 74048, 76889, 14429, 93708, 23912, 258604, 14360, 14365, 14701, 268934, 110855	0.087	1.321
GOTERM_MF_DIRECT	GO:0046872~metal ion binding	30	20.00	107823, 100233208, 100041433, 20182, 70081, 241073, 17764, 72043, 18570, 106042, 16876, 170638, 170753, 104099, 67785, 50770, 68350, 14681, 18105, 19415, 13518, 433520, 110593, 112415, 22380, 56490, 328274, 14360, 433791, 110855	0.089	1.311
GOTERM_CC_DIRECT	GO:0030424~axon	6	4.00	16572, 13838, 75785, 14365, 68350, 22352	0.092	2.491
GOTERM_MF_DIRECT	GO:0032403~protein complex binding	6	4.00	16572, 56693, 14360, 19128, 22241, 14681	0.096	2.457
UP_SEQ_FEATURE	domain:Protein kinase	7	4.67	14254, 13838, 76889, 27263, 24013, 14360, 22241	0.096	2.203
GOTERM_BP_DIRECT	GO:0008104~protein localization	3	2.00	16572, 235633, 22241	0.097	5.675
UP_SEQ_FEATURE	lipid moiety-binding region:S-palmitoyl cysteine	4	2.67	14429, 67168, 14360, 14681	0.103	3.531
KEGG_PATHWAY	mmu04144:Endocytosis	5	3.33	72685, 16572, 14254, 24013, 225861	0.110	2.670
UP_KEYWORDS	Palmitate	5	3.33	14429, 67168, 14360, 13518, 14681	0.111	2.723
GOTERM_BP_DIRECT	GO:0030900~forebrain development	3	2.00	18223, 14360, 14681	0.112	5.213

Supplemental File 4. Continuation.

Category	Term	Count	%	Genes	PValue	Fold Enrichment
GOTERM_BP_DIRECT	GO:0016310~phosphorylation	8	5.33	14254, 13838, 76889, 27263, 24013, 14360, 22241, 233011	0.113	1.953
UP_KEYWORDS	Disulfide bond	25	16.67	14254, 257630, 14172, 108115, 73382, 52020, 18208, 60505, 106347, 67374, 12164, 74048, 76943, 214523, 14429, 67168, 228413, 50493, 104099, 19128, 12519, 22421, 14365, 85031, 268934	0.119	1.325
KEGG_PATHWAY	mmu05200:Pathways in cancer	6	4.00	14172, 67168, 22421, 14365, 20182, 14701	0.120	2.244
INTERPRO	IPR011009:Protein kinase-like domain	7	4.67	14254, 13838, 76889, 27263, 24013, 14360, 22241	0.126	2.042
GOTERM_MF_DIRECT	GO:0031625~ubiquitin protein ligase binding	5	3.33	142687, 20747, 55943, 68350, 15526	0.127	2.581
UP_KEYWORDS	SH3 domain	4	2.67	52906, 14360, 320664, 13518	0.130	3.184
GOTERM_MF_DIRECT	GO:0003676~nucleic acid binding	13	8.67	110593, 112415, 22380, 16549, 56490, 20382, 328274, 100233208, 100041433, 70081, 433791, 433520, 17764	0.131	1.541
GOTERM_BP_DIRECT	GO:0001503~ossification	3	2.00	12164, 13406, 14172	0.134	4.670
KEGG_PATHWAY	mmu04916:Melanogenesis	3	2.00	22421, 14365, 14681	0.139	4.499
UP_SEQ_FEATURE	nucleotide phosphate-binding region:GTP	5	3.33	19342, 74222, 74211, 23912, 14681	0.141	2.476
GOTERM_CC_DIRECT	GO:0044297~cell body	3	2.00	13838, 14681, 22352	0.141	4.518
UP_SEQ_FEATURE	domain:Ig-like V-type	3	2.00	12519, 106347, 67374	0.147	4.389
GOTERM_CC_DIRECT	GO:0005886~plasma membrane	38	25.33	14254, 19265, 52020, 628185, 241073, 67374, 72043, 19342, 329251, 13838, 20499, 259111, 12695, 67168, 258713, 227059, 50770, 63959, 14681, 19415, 30791, 331374, 108115, 13518, 106347, 22352, 74048, 22380, 76889, 14429, 93708, 23912, 258604, 14360, 14365, 14701, 110855, 268934	0.151	1.198
UP_SEQ_FEATURE	lipid moiety-binding region:N-myristoyl glycine	3	2.00	170638, 14360, 14681	0.152	4.309
UP_SEQ_FEATURE	disulfide bond	21	14.00	14254, 257630, 73382, 52020, 18208, 60505, 106347, 67374, 12164, 19342, 74048, 76943, 214523, 14429, 67168, 228413, 50493, 19128, 12519, 14365, 85031	0.153	1.322
KEGG_PATHWAY	mmu04510:Focal adhesion	4	2.67	329251, 14254, 104099, 14360	0.156	2.869
UP_SEQ_FEATURE	binding site:ATP	7	4.67	14254, 13838, 27263, 24013, 14360, 22241, 233011	0.160	1.897
KEGG_PATHWAY	mmu04810:Regulation of actin cytoskeleton	4	2.67	329251, 14172, 104099, 14701	0.167	2.775
UP_KEYWORDS	Extracellular matrix	4	2.67	13406, 56693, 22421, 18208	0.168	2.818
KEGG_PATHWAY	mmu04725:Cholinergic synapse	3	2.00	14360, 14681, 14701	0.171	3.941
UP_SEQ_FEATURE	compositionally biased region:Poly-Pro	5	3.33	13406, 228413, 20747, 57752, 541610	0.172	2.290
GOTERM_MF_DIRECT	GO:0016301~kinase activity	8	5.33	14254, 13838, 76889, 27263, 24013, 14360, 22241, 233011	0.172	1.740
KEGG_PATHWAY	mmu04724:Glutamatergic synapse	3	2.00	14681, 14701, 268934	0.176	3.873
GOTERM_MF_DIRECT	GO:0005125~cytokine activity	4	2.67	12164, 257630, 22421, 60505	0.177	2.740
GOTERM_BP_DIRECT	GO:0008283~cell proliferation	4	2.67	16876, 14172, 50493, 57752	0.180	2.717
GOTERM_MF_DIRECT	GO:0005215~transporter activity	4	2.67	228993, 15247, 20499, 108115	0.182	2.702
UP_SEQ_FEATURE	topological domain:Cytoplasmic	23	15.33	14254, 103963, 52020, 55943, 106347, 67374, 74048, 15247, 13838, 214523, 20499, 14429, 67168, 228413, 74488, 12519, 50770, 63959, 14365, 68350, 268934, 215090, 30791	0.187	1.262
UP_SEQ_FEATURE	propeptide:Removed in mature form	4	2.67	24013, 23912, 14701, 110855	0.188	2.655
GOTERM_MF_DIRECT	GO:0005509~calcium ion binding	8	5.33	18570, 170638, 93708, 228413, 19128, 52020, 13518, 72043	0.195	1.678
UP_KEYWORDS	ATP-binding	12	8.00	16572, 14254, 13838, 76889, 331374, 27263, 24013, 14360, 22241, 50770, 233011, 15526	0.195	1.457
UP_KEYWORDS	Cytoskeleton	10	6.67	74222, 78748, 16572, 21916, 23912, 52906, 320664, 57752, 13518, 72776	0.196	1.543
GOTERM_BP_DIRECT	GO:0001764~neuron migration	3	2.00	14360, 14365, 18208	0.199	3.615
KEGG_PATHWAY	mmu04151:PI3K-Akt signaling pathway	5	3.33	14254, 14172, 67168, 104099, 14701	0.201	2.115
GOTERM_CC_DIRECT	GO:0014069~postsynaptic density	4	2.67	72685, 13838, 14360, 13518	0.201	2.571
KEGG_PATHWAY	mmu04360:Axon guidance	3	2.00	13838, 14360, 18208	0.209	3.453
GOTERM_BP_DIRECT	GO:0001525~angiogenesis	4	2.67	18570, 14254, 14172, 22421	0.212	2.501
GOTERM_BP_DIRECT	GO:0098779~mitophagy in response to mitochondrial depolarization	3	2.00	241589, 22241, 233011	0.218	3.396
UP_KEYWORDS	Methylation	9	6.00	19342, 106042, 16549, 24013, 23912, 20182, 14701, 110855, 15526	0.219	1.552
KEGG_PATHWAY	mmu04728:Dopaminergic synapse	3	2.00	16572, 14681, 14701	0.222	3.324
INTERPRO	IPR017441:Protein kinase, ATP binding site	5	3.33	14254, 13838, 24013, 14360, 22241	0.222	2.058
GOTERM_BP_DIRECT	GO:0055085~transmembrane transport	5	3.33	228993, 15247, 20499, 227059, 30791	0.223	2.053
UP_KEYWORDS	Calcium	8	5.33	18570, 170638, 93708, 104099, 19128, 52020, 13518, 72043	0.229	1.601
UP_SEQ_FEATURE	compositionally biased region:Ser-rich	5	3.33	78748, 170753, 74042, 320664, 541610	0.229	2.026
INTERPRO	IPR011992:EF-hand-like domain	4	2.67	18570, 170638, 13518, 241073	0.234	2.376
KEGG_PATHWAY	mmu04310:Wnt signaling pathway	3	2.00	106042, 22421, 14365	0.239	3.159
GOTERM_BP_DIRECT	GO:0007399~nervous system development	5	3.33	13838, 14172, 18223, 14365, 17135	0.241	1.982
UP_SEQ_FEATURE	topological domain:Extracellular	18	12.00	14254, 52020, 106347, 67374, 74048, 15247, 13838, 214523, 14429, 67168, 228413, 74488, 12519, 50770, 14365, 63959, 268934, 30791	0.246	1.261
GOTERM_CC_DIRECT	GO:0005925~focal adhesion	5	3.33	14254, 320664, 13518, 22352, 15526	0.247	1.964
GOTERM_CC_DIRECT	GO:0043025~neuronal cell body	6	4.00	16572, 16549, 50493, 22241, 14365, 68350	0.263	1.726
GOTERM_CC_DIRECT	GO:0016020~membrane	50	33.33	103963, 73382, 55943, 19342, 18570, 16549, 67168, 104099, 227059, 63959, 68350, 14681, 30791, 16572, 108115, 110265, 15247, 622554, 12519, 228993, 14254, 19265, 21916, 52020, 241073, 67374, 106042, 13838, 214523, 20499, 12695, 24013, 74488, 22241, 50770, 71844, 13518, 106347, 74048, 76889, 93708, 228413, 23912, 14360, 14365, 68510, 14701, 215090, 268934, 110855	0.268	1.098
UP_KEYWORDS	mRNA processing	4	2.67	22380, 16549, 20382, 67337	0.280	2.157
UP_SEQ_FEATURE	active site:Proton acceptor	7	4.67	14254, 13838, 27263, 24013, 50493, 14360, 22241	0.287	1.558
GOTERM_CC_DIRECT	GO:0043005~neuron projection	5	3.33	16572, 22241, 14681, 268934, 22352	0.288	1.829
KEGG_PATHWAY	mmu04514:Cell adhesion molecules (CAMs)	3	2.00	104099, 12519, 67374	0.290	2.749
GOTERM_CC_DIRECT	GO:0005856~cytoskeleton	10	6.67	74222, 78748, 21916, 23912, 52906, 320664, 57752, 13518, 72776, 22352	0.292	1.380
UP_KEYWORDS	Cell adhesion	5	3.33	13838, 93708, 104099, 320664, 13518	0.297	1.803
KEGG_PATHWAY	mmu05202:Transcriptional misregulation in cancer	3	2.00	14254, 107823, 20182	0.300	2.683
GOTERM_MF_DIRECT	GO:0008270~zinc ion binding	10	6.67	106042, 107823, 66609, 16876, 22380, 74042, 67785, 68350, 20182, 241073	0.303	1.364
REACTOME_PATHWAY	R-MMU-72163:R-MMU-72163	3	2.00	22380, 20382, 67337	0.317	2.576
GOTERM_CC_DIRECT	GO:0030054~cell junction	7	4.67	13838, 15424, 12695, 52906, 320664, 13518, 67374	0.319	1.498

Supplemental File 4. Continuation.

Category	Term	Count	%	Genes	PValue	Fold Enrichment
UP_KEYWORDS	GTP-binding	4	2.67	19342, 74222, 23912, 14681	0.321	1.995
GOTERM_CC_DIRECT	GO:0005578~proteinaceous extracellular matrix	4	2.67	13406, 56693, 22421, 18208	0.335	1.944
UP_KEYWORDS	Cytokine	3	2.00	12164, 257630, 60505	0.338	2.483
UP_KEYWORDS	Oxidoreductase	6	4.00	66609, 74042, 13086, 50493, 110265, 18105	0.338	1.554
GOTERM_CC_DIRECT	GO:0005576~extracellular region	14	9.33	257630, 14172, 52020, 60505, 18208, 12164, 13406, 76943, 56693, 228413, 19128, 22421, 85031, 629761	0.343	1.227
UP_KEYWORDS	Isopeptide bond	8	5.33	16549, 56490, 103963, 20957, 67785, 13518, 68350, 22352	0.347	1.390
GOTERM_BP_DIRECT	GO:0006397~mRNA processing	4	2.67	22380, 16549, 20382, 67337	0.361	1.856
UP_KEYWORDS	Cell junction	6	4.00	13838, 12695, 52906, 320664, 13518, 67374	0.364	1.503
UP_SEQ_FEATURE	transmembrane region	30	20.00	228993, 14254, 19265, 103963, 73382, 55943, 52020, 67374, 13838, 20499, 214523, 67168, 74488, 227059, 50770, 63959, 68350, 30791, 108115, 106347, 74048, 15247, 76889, 14429, 228413, 12519, 14365, 68510, 215090, 268934	0.368	1.099
GOTERM_CC_DIRECT	GO:0005654~nucleoplasm	15	10.00	110265, 17764, 17135, 215351, 15424, 22380, 16549, 56490, 70461, 50493, 20957, 67785, 68350, 18105, 233011	0.369	1.191
UP_SEQ_FEATURE	active site:Charge relay system	3	2.00	214523, 73382, 85031	0.369	2.312
UP_KEYWORDS	Developmental protein	8	5.33	12164, 14254, 13838, 15424, 18223, 14360, 22421, 14365	0.369	1.357
INTERPRO	IPR001452:Src homology-3 domain	3	2.00	52906, 14360, 320664	0.373	2.295
GOTERM_CC_DIRECT	GO:0015629~actin cytoskeleton	3	2.00	329251, 110265, 13518	0.373	2.293
GOTERM_BP_DIRECT	GO:0007275~multicellular organism development	9	6.00	12164, 14254, 13838, 15424, 18223, 14360, 67785, 22421, 14365	0.375	1.307
GOTERM_MF_DIRECT	GO:0004842~ubiquitin-protein transferase activity	4	2.67	142687, 230590, 75785, 68350	0.379	1.799
GOTERM_BP_DIRECT	GO:0042981~regulation of apoptotic process	3	2.00	12164, 14360, 52020	0.381	2.253
GOTERM_CC_DIRECT	GO:0016607~nuclear speck	3	2.00	22380, 20382, 20747	0.383	2.248
UP_KEYWORDS	Coiled coil	21	14.00	16572, 55943, 13518, 72776, 241073, 72043, 17135, 22352, 78748, 74222, 67434, 329251, 110593, 329366, 12695, 18223, 20957, 50770, 57752, 225861, 71844	0.385	1.130
SMART	SM00326:SH3	3	2.00	52906, 14360, 320664	0.389	2.205
KEGG_PATHWAY	mmu05205:Proteoglycans in cancer	3	2.00	329251, 22421, 14365	0.390	2.194
GOTERM_MF_DIRECT	GO:0008017~microtubule binding	3	2.00	16572, 329366, 13518	0.393	2.199
UP_KEYWORDS	Membrane	55	36.67	103963, 73382, 55943, 628185, 19342, 18570, 259111, 67168, 13086, 104099, 227059, 63959, 68350, 14681, 30791, 319293, 108115, 110265, 15247, 622554, 12519, 228993, 14254, 19265, 52020, 241073, 67374, 106042, 13838, 214523, 20499, 12695, 258713, 24013, 241589, 74488, 19128, 50770, 71844, 13518, 106347, 74048, 14429, 76889, 93708, 23912, 228413, 14360, 258604, 14365, 68510, 14701, 110855, 215090, 268934	0.398	1.049
UP_SEQ_FEATURE	nucleotide phosphate-binding region:ATP	8	5.33	16572, 14254, 13838, 27263, 24013, 14360, 22241, 233011	0.400	1.313
GOTERM_BP_DIRECT	GO:0007155~cell adhesion	5	3.33	13838, 93708, 104099, 320664, 13518	0.402	1.541
GOTERM_BP_DIRECT	GO:0016192~vesicle-mediated transport	3	2.00	16572, 52906, 55943	0.414	2.105
KEGG_PATHWAY	mmu04015:Rap1 signaling pathway	3	2.00	14254, 14172, 14681	0.415	2.081
UP_KEYWORDS	Ubl conjugation	11	7.33	14254, 16549, 56490, 103963, 67785, 55943, 14365, 13518, 68350, 72776, 22352	0.416	1.210
GOTERM_MF_DIRECT	GO:0042802~identical protein binding	6	4.00	18570, 14254, 235633, 52906, 68350, 22352	0.416	1.407
UP_KEYWORDS	mRNA splicing	3	2.00	22380, 16549, 20382	0.423	2.069
GOTERM_BP_DIRECT	GO:0007166~cell surface receptor signaling pathway	3	2.00	257630, 14360, 14365	0.428	2.047
GOTERM_CC_DIRECT	GO:0005769~early endosome	3	2.00	78670, 55943, 50770	0.430	2.039
UP_KEYWORDS	Secreted	12	8.00	12164, 13406, 76943, 56693, 257630, 14172, 19128, 22421, 60505, 85031, 18208, 629761	0.430	1.179
UP_KEYWORDS	Neurogenesis	3	2.00	13838, 18223, 14365	0.437	2.011
GOTERM_MF_DIRECT	GO:0000166~nucleotide binding	15	10.00	14254, 74222, 19342, 13838, 27263, 20382, 24013, 23912, 14360, 22241, 50770, 14681, 233011, 110855, 15526	0.438	1.136
GOTERM_BP_DIRECT	GO:0006355~regulation of transcription, DNA-templated	17	11.33	107823, 100233208, 100041433, 20182, 70081, 433520, 17764, 17135, 110593, 15424, 112415, 16876, 16549, 56490, 70461, 328274, 433791	0.445	1.115
GOTERM_MF_DIRECT	GO:0004871~signal transducer activity	6	4.00	67168, 14365, 68350, 14681, 14701, 268934	0.447	1.357
INTERPRO	IPR011993:Pleckstrin homology-like domain	4	2.67	331374, 18223, 78670, 19415	0.458	1.586
GOTERM_CC_DIRECT	GO:0005730~nucleolus	7	4.67	13406, 14172, 74042, 50493, 59028, 57752, 15526	0.462	1.277
GOTERM_CC_DIRECT	GO:0048471~perinuclear region of cytoplasm	6	4.00	19342, 16572, 12695, 55943, 13518, 22352	0.464	1.332
UP_KEYWORDS	Nucleus	29	19.33	107823, 20747, 59028, 20182, 17764, 17135, 18570, 13406, 106042, 15424, 16876, 16549, 20382, 20957, 71844, 67337, 15526, 110265, 13518, 215351, 22380, 56490, 622554, 70461, 74042, 14360, 57752, 68510, 233011	0.465	1.059
UP_KEYWORDS	Transport	13	8.67	19342, 228993, 15247, 20499, 16549, 108115, 227059, 55943, 50770, 63959, 225861, 71844, 30791	0.470	1.132
GOTERM_BP_DIRECT	GO:0008380~RNA splicing	3	2.00	22380, 16549, 20382	0.477	1.860
INTERPRO	IPR001849:Pleckstrin homology domain	3	2.00	331374, 78670, 19415	0.478	1.857
GOTERM_MF_DIRECT	GO:0005525~GTP binding	4	2.67	19342, 74222, 23912, 14681	0.481	1.531
SMART	SM00220:S_TKc	4	2.67	13838, 27263, 24013, 22241	0.482	1.524
GOTERM_BP_DIRECT	GO:0008284~positive regulation of cell proliferation	5	3.33	14172, 100040854, 22421, 60505, 18208	0.486	1.379
UP_SEQ_FEATURE	compositionally biased region:Pro-rich	6	4.00	72685, 170753, 50493, 17764, 215090, 69871	0.487	1.297
UP_KEYWORDS	DNA-binding	11	7.33	67434, 15424, 107823, 16876, 16549, 56490, 622554, 20957, 20182, 17764, 17135	0.497	1.135
GOTERM_BP_DIRECT	GO:0035556~intracellular signal transduction	4	2.67	142687, 331374, 27263, 19415	0.498	1.494
UP_KEYWORDS	Endosome	4	2.67	18570, 14254, 13838, 50770	0.498	1.495
UP_KEYWORDS	Receptor	17	11.33	19265, 14254, 20182, 106347, 628185, 74048, 13838, 14429, 259111, 67168, 258713, 24013, 104099, 258604, 12519, 14365, 268934	0.504	1.077
SMART	SM00233:PH	3	2.00	331374, 78670, 19415	0.517	1.717
GOTERM_BP_DIRECT	GO:0043066~negative regulation of apoptotic process	5	3.33	100040854, 24013, 52906, 22421, 68510	0.521	1.320
GOTERM_CC_DIRECT	GO:0005634~nucleus	40	26.67	14254, 21916, 107823, 14172, 66399, 20747, 59028, 20182, 17764, 17135, 13406, 18570, 106042, 142687, 15424, 16876, 27263, 16549, 170753, 20382, 20957, 71844, 67337, 15526, 217379, 68222, 110265, 13518, 215351, 110593, 22380, 56490, 74042, 70461, 622554, 50493, 14360, 57752, 233011, 68510	0.524	1.021
UP_KEYWORDS	Ubl conjugation pathway	5	3.33	215351, 18223, 230590, 20747, 68350	0.526	1.312

Supplemental File 4. Continuation.

Category	Term	Count	%	Genes	PValue	Fold Enrichment
GOTERM_MF_DIRECT	GO:0005102~receptor binding	4	2.67	14172, 14360, 22421, 30791	0.530	1.423
UP_KEYWORDS	Transferase	11	7.33	14254, 13838, 107823, 76889, 331374, 27263, 24013, 103963, 14360, 22241, 233011	0.537	1.101
GOTERM_BP_DIRECT	GO:0045892~negative regulation of transcription, DNA-templated	5	3.33	106042, 16876, 100040854, 56490, 223453	0.539	1.290
KEGG_PATHWAY	mmu05166:HTLV-I infection	3	2.00	70461, 22421, 14365	0.553	1.602
GOTERM_BP_DIRECT	GO:0006810~transport	13	8.67	19342, 228993, 15247, 20499, 16549, 108115, 227059, 55943, 50770, 63959, 225861, 71844, 30791	0.556	1.066
GOTERM_MF_DIRECT	GO:0004674~protein serine/threonine kinase activity	4	2.67	76889, 27263, 24013, 22241	0.556	1.370
UP_KEYWORDS	Apoptosis	4	2.67	18570, 18208, 68350, 223453	0.564	1.354
GOTERM_MF_DIRECT	GO:0019901~protein kinase binding	4	2.67	329251, 20499, 22241, 22352	0.566	1.351
KEGG_PATHWAY	mmu04080:Neuroactive ligand-receptor interaction	3	2.00	14429, 67168, 268934	0.567	1.563
GOTERM_CC_DIRECT	GO:0031012~extracellular matrix	3	2.00	103963, 22352, 15526	0.569	1.567
UP_KEYWORDS	Cytoplasm	27	18.00	21916, 320664, 75785, 72776, 78748, 13406, 329251, 106042, 16549, 12695, 18223, 235633, 52906, 22241, 18105, 16572, 110265, 13518, 22352, 74222, 76889, 70461, 23912, 50493, 14360, 57752, 233011	0.570	1.015
GOTERM_MF_DIRECT	GO:0005524~ATP binding	11	7.33	16572, 14254, 13838, 331374, 27263, 24013, 14360, 22241, 50770, 233011, 15526	0.573	1.070
GOTERM_CC_DIRECT	GO:0009986~cell surface	5	3.33	13838, 12519, 22421, 14365, 72043	0.583	1.221
GOTERM_CC_DIRECT	GO:0005737~cytoplasm	43	28.67	21916, 14172, 20747, 75785, 320664, 18208, 72776, 13406, 78748, 18570, 329251, 74211, 106042, 142687, 13838, 12695, 16549, 27263, 18223, 235633, 52906, 74488, 67785, 22241, 68350, 18105, 19415, 15526, 16572, 110265, 13518, 22352, 215351, 74222, 76943, 76889, 70461, 50493, 23912, 14360, 57752, 14365, 233011	0.595	0.996
GOTERM_CC_DIRECT	GO:0005887~integral component of plasma membrane	8	5.33	14254, 13838, 20499, 67168, 108115, 227059, 63959, 268934	0.596	1.091
GOTERM_MF_DIRECT	GO:0003677~DNA binding	13	8.67	107823, 20182, 17135, 17764, 67434, 110593, 15424, 16876, 16549, 56490, 622554, 20957, 67785	0.603	1.032
INTERPRO	IPR008271:Serine/threonine-protein kinase, active site	3	2.00	27263, 24013, 22241	0.607	1.461
GOTERM_BP_DIRECT	GO:0010468~regulation of gene expression	3	2.00	217379, 52020, 22241	0.609	1.456
GOTERM_CC_DIRECT	GO:0009897~external side of plasma membrane	3	2.00	74048, 12519, 52020	0.610	1.454
GOTERM_CC_DIRECT	GO:0030425~dendrite	4	2.67	13838, 16549, 14365, 14681	0.617	1.254
GOTERM_MF_DIRECT	GO:0043565~sequence-specific DNA binding	5	3.33	15424, 107823, 16876, 20182, 17135	0.625	1.158
GOTERM_CC_DIRECT	GO:0016324~apical plasma membrane	3	2.00	12695, 14365, 63959	0.628	1.405
GOTERM_CC_DIRECT	GO:0005615~extracellular space	10	6.67	12164, 76943, 14254, 56693, 257630, 19128, 52020, 22421, 60505, 72043	0.643	1.021
GOTERM_BP_DIRECT	GO:0045944~positive regulation of transcription from RNA polymerase II promoter	7	4.67	14429, 257630, 70461, 52906, 22421, 20182, 17764	0.653	1.051
GOTERM_CC_DIRECT	GO:0005694~chromosome	3	2.00	107823, 622554, 20957	0.654	1.340
UP_KEYWORDS	Transmembrane	41	27.33	228993, 14254, 19265, 103963, 73382, 55943, 52020, 628185, 67374, 13838, 20499, 214523, 259111, 258713, 67168, 241589, 104099, 19128, 74488, 227059, 50770, 63959, 68350, 30791, 319293, 108115, 13518, 106347, 74048, 15247, 14429, 76889, 93708, 622554, 228413, 258604, 12519, 14365, 68510, 215090, 268934	0.656	0.976
GOTERM_BP_DIRECT	GO:0042493~response to drug	3	2.00	14360, 14365, 14681	0.661	1.322
INTERPRO	IPR013783:Immunoglobulin-like fold	7	4.67	74048, 14254, 13838, 12519, 52020, 106347, 67374	0.670	1.033
GOTERM_MF_DIRECT	GO:0016740~transferase activity	10	6.67	14254, 13838, 107823, 76889, 27263, 24013, 103963, 14360, 22241, 233011	0.673	0.996
GOTERM_CC_DIRECT	GO:0042995~cell projection	5	3.33	13838, 52906, 75785, 13518, 22352	0.680	1.079
GOTERM_BP_DIRECT	GO:0016567~protein ubiquitination	3	2.00	142687, 75785, 68350	0.696	1.238
UP_KEYWORDS	Serine/threonine-protein kinase	3	2.00	27263, 24013, 22241	0.701	1.226
UP_KEYWORDS	Chromosome	3	2.00	107823, 622554, 20957	0.707	1.214
UP_KEYWORDS	Transmembrane helix	40	26.67	228993, 14254, 19265, 103963, 73382, 55943, 52020, 628185, 67374, 13838, 20499, 214523, 259111, 258713, 67168, 241589, 104099, 19128, 74488, 227059, 50770, 63959, 68350, 30791, 319293, 108115, 106347, 74048, 15247, 14429, 76889, 93708, 622554, 228413, 258604, 12519, 14365, 68510, 215090, 268934	0.716	0.954
UP_KEYWORDS	Endoplasmic reticulum	6	4.00	18570, 103963, 13086, 50770, 13518, 72043	0.719	0.996
UP_KEYWORDS	Ion transport	4	2.67	20499, 108115, 227059, 30791	0.721	1.070
GOTERM_CC_DIRECT	GO:0005829~cytosol	11	7.33	78748, 106042, 66609, 16549, 217379, 50493, 14360, 22241, 55943, 19415, 22352	0.726	0.947
UP_KEYWORDS	Cell cycle	4	2.67	74222, 74042, 20957, 72776	0.728	1.058
GOTERM_BP_DIRECT	GO:0006915~apoptotic process	4	2.67	18570, 18208, 68350, 223453	0.734	1.049
GOTERM_MF_DIRECT	GO:0019899~enzyme binding	3	2.00	13086, 14360, 15526	0.737	1.145
GOTERM_BP_DIRECT	GO:0010628~positive regulation of gene expression	3	2.00	52906, 22421, 22352	0.746	1.124
UP_KEYWORDS	Differentiation	4	2.67	12164, 14254, 52906, 19415	0.748	1.025
GOTERM_BP_DIRECT	GO:0006811~ion transport	4	2.67	20499, 108115, 227059, 30791	0.749	1.024
GOTERM_BP_DIRECT	GO:0007283~spermatogenesis	3	2.00	12164, 56693, 20957	0.756	1.102
GOTERM_CC_DIRECT	GO:0005783~endoplasmic reticulum	8	5.33	18570, 13838, 56693, 217379, 103963, 50770, 13518, 72043	0.759	0.929
INTERPRO	IPR013083:Zinc finger, RING/FYVE/PHD-type	3	2.00	107823, 74042, 68350	0.760	1.093
UP_KEYWORDS	Transducer	10	6.67	14429, 259111, 67168, 258713, 258604, 14365, 14681, 628185, 14701, 268934	0.761	0.919
GOTERM_CC_DIRECT	GO:0005813~centrosome	3	2.00	329366, 52906, 72776	0.775	1.059
GOTERM_CC_DIRECT	GO:0043234~protein complex	4	2.67	12695, 19128, 71844, 14681	0.776	0.978
UP_KEYWORDS	Cell projection	4	2.67	13838, 52906, 75785, 13518	0.777	0.977
UP_KEYWORDS	Hydrolase	9	6.00	72685, 215351, 214523, 73382, 50770, 85031, 110855, 72043, 215090	0.779	0.905
GOTERM_MF_DIRECT	GO:0016491~oxidoreductase activity	4	2.67	66609, 50493, 110265, 18105	0.780	0.971
GOTERM_CC_DIRECT	GO:0031410~cytoplasmic vesicle	4	2.67	18570, 235633, 13518, 268934	0.792	0.951
GOTERM_MF_DIRECT	GO:0042803~protein homodimerization activity	5	3.33	18570, 257630, 50493, 13518, 20182	0.794	0.919
INTERPRO	IPR027417:P-loop containing nucleoside triphosphate hydrolase	5	3.33	19342, 74222, 16572, 23912, 14681	0.812	0.892
UP_KEYWORDS	Transit peptide	3	2.00	66399, 110265, 15526	0.814	0.974
GOTERM_MF_DIRECT	GO:0005515~protein binding	25	16.67	16572, 20747, 13518, 18208, 20182, 17135, 22352, 18570, 74048, 106042, 13838, 16876, 22380, 12695, 235633, 20382, 52906, 14360, 20957, 12519, 22241, 22421, 14365, 14681, 15526	0.818	0.896
GOTERM_CC_DIRECT	GO:0016021~integral component of membrane	41	27.33	228993, 14254, 19265, 103963, 73382, 55943, 52020, 628185, 67374, 13838, 20499, 214523, 259111, 258713, 67168, 241589, 104099, 19128, 74488, 227059, 50770, 63959, 68350, 30791, 319293, 108115, 13518, 106347, 74048, 15247, 14429, 76889, 93708, 622554, 228413, 258604, 12519, 14365, 68510, 215090, 268934	0.821	0.916
GOTERM_MF_DIRECT	GO:0016787~hydrolase activity	9	6.00	72685, 215351, 214523, 73382, 50770, 85031, 110855, 72043, 215090	0.824	0.861
GOTERM_BP_DIRECT	GO:0055114~oxidation-reduction process	4	2.67	66609, 50493, 110265, 18105	0.831	0.884
GOTERM_CC_DIRECT	GO:0070062~extracellular exosome	15	10.00	110265, 22352, 18570, 212114, 12695, 93708, 20382, 23912, 50493, 74488, 19128, 85031, 18105, 14701, 15526	0.836	0.862
GOTERM_CC_DIRECT	GO:0005789~endoplasmic reticulum membrane	4	2.67	18570, 103963, 13086, 13518	0.842	0.865

Supplemental File 4. Continuation.

Category	Term	Count	%	Genes	PValue	Fold Enrichment
GOTERM_MF_DIRECT	GO:0003700~transcription factor activity, sequence-specific DNA binding	5	3.33	15424, 112415, 20182, 17764, 17135	0.854	0.830
UP_KEYWORDS	Acetylation	16	10.67	16572, 103963, 110265, 17764, 22352, 18570, 19342, 74211, 16549, 20382, 23912, 67785, 68510, 14701, 223453, 15526	0.856	0.848
INTERPRO	IPR013106:Immunoglobulin V-set	3	2.00	74048, 12519, 67374	0.856	0.878
UP_KEYWORDS	Protein transport	3	2.00	19342, 225861, 71844	0.864	0.861
GOTERM_MF_DIRECT	GO:0044822~poly(A) RNA binding	6	4.00	16549, 103963, 66399, 20382, 67337, 15526	0.879	0.790
UP_KEYWORDS	Mitochondrion	5	3.33	76889, 66399, 110265, 68350, 15526	0.884	0.782
UP_KEYWORDS	Activator	3	2.00	70461, 74042, 17764	0.892	0.796
GOTERM_BP_DIRECT	GO:0045893~positive regulation of transcription, DNA-templated	3	2.00	74042, 22421, 17764	0.899	0.778
UP_KEYWORDS	G-protein coupled receptor	8	5.33	14429, 259111, 67168, 258713, 258604, 14365, 628185, 268934	0.905	0.761
GOTERM_BP_DIRECT	GO:0015031~protein transport	3	2.00	19342, 225861, 71844	0.908	0.757
GOTERM_BP_DIRECT	GO:0007049~cell cycle	3	2.00	74222, 20957, 72776	0.918	0.730
GOTERM_CC_DIRECT	GO:0005739~mitochondrion	8	5.33	19342, 76889, 66399, 50493, 14360, 110265, 68350, 15526	0.935	0.714
GOTERM_BP_DIRECT	GO:0007186~G-protein coupled receptor signaling pathway	8	5.33	259111, 67168, 258713, 258604, 14365, 14681, 14701, 268934	0.943	0.701
INTERPRO	IPR000276:G protein-coupled receptor, rhodopsin-like	6	4.00	14429, 259111, 67168, 319293, 258713, 258604	0.949	0.667
GOTERM_BP_DIRECT	GO:0000122~negative regulation of transcription from RNA polymerase II promoter	3	2.00	107823, 56490, 20747	0.957	0.615
GOTERM_CC_DIRECT	GO:0043231~intracellular membrane-bounded organelle	3	2.00	13406, 14360, 50770	0.957	0.614
GOTERM_MF_DIRECT	GO:0004930~G-protein coupled receptor activity	8	5.33	259111, 67168, 319293, 258713, 258604, 14365, 628185, 268934	0.958	0.671
UP_KEYWORDS	Sensory transduction	5	3.33	259111, 258713, 24013, 258604, 110855	0.960	0.627
GOTERM_BP_DIRECT	GO:0030154~cell differentiation	3	2.00	12164, 14254, 19415	0.968	0.575
GOTERM_MF_DIRECT	GO:0003723~RNA binding	3	2.00	16549, 20382, 59028	0.971	0.564
INTERPRO	IPR017452:GPCR, rhodopsin-like, 7TM	6	4.00	14429, 259111, 67168, 319293, 258713, 258604	0.982	0.570
KEGG_PATHWAY	mmu01100:Metabolic pathways	4	2.67	331374, 103963, 13086, 233011	0.994	0.465
GOTERM_CC_DIRECT	GO:0005794~Golgi apparatus	3	2.00	13838, 72043, 215090	0.997	0.387

Supplemental File 5.

Supplemental Methods:

Western Blot Analysis

Cytoplasmic and nuclear protein fractions were extracted from WT and frozen cells (~10 x 10^6) using the NE-PER kit (Pierce Biotechnology, Rockford, IL) supplemented with phosphatase inhibitors (Pierce Biotechnology, Rockford, IL) and protease inhibitors (Roche Diagnostics, Indianapolis, IN). Protein extracts (40 µg) were separated on a precast 4-20% SDSpage gel (BioRad Technologies Inc, Hercules, CA) and transferred to nitrocellulose membranes using the iBlot 2 transfer system (Life Technologies, Carlsbad, CA). The membranes were blocked for 1 hour in 5% milk dissolved in Tris buffered saline containing 0.1% Tween-20 to deter non-specific binding and immunoblotted with 10 mL of diluted primary antibodies as follows: Rabbit polyclonal antibody to SC35 (GeneTex, Irvine, CA) was diluted at 1:500, Rabbit polyclonal antibody to TrkA (Cell Signaling Technology Inc., Danvers, MA) was diluted at 1:200; Rabbit polyclonal antibody to beta-Tubulin (Abcam, Cambridge, MA) was diluted at 1:500. Antibodies were diluted in 1% milk dissolved in Tris buffered saline containing 0.1% Tween-20 and incubated on a rocking table for 24 hours at 4°C. Goat anti-rabbit conjugated IgG-HRP antibody (Jackson ImmunoResearch Laboratories, Inc, West Grove, PA) was used against all primary antibodies. Secondary antibody was diluted 1:4,000 in 1% milk dissolved in Tris buffered saline containing 0.1% Tween-20 and incubated on a rocking table for 1 hour at room temperature. Proteins were visualized by SuperSignal™ West Pico chemiluminescent substrate and monitored using Biospectrum 810 UVP imaging system (UVP LLC, Upland, CA). Densitometry analysis of protein bands was performed using ImageJ software (National Institutes of Health [NIH], http://rsb.info.nih.gov/ij/). Bands corresponding to SC35 and TRKA were normalized to the density of respective β -Tubulin bands.

Supplemental File 6.

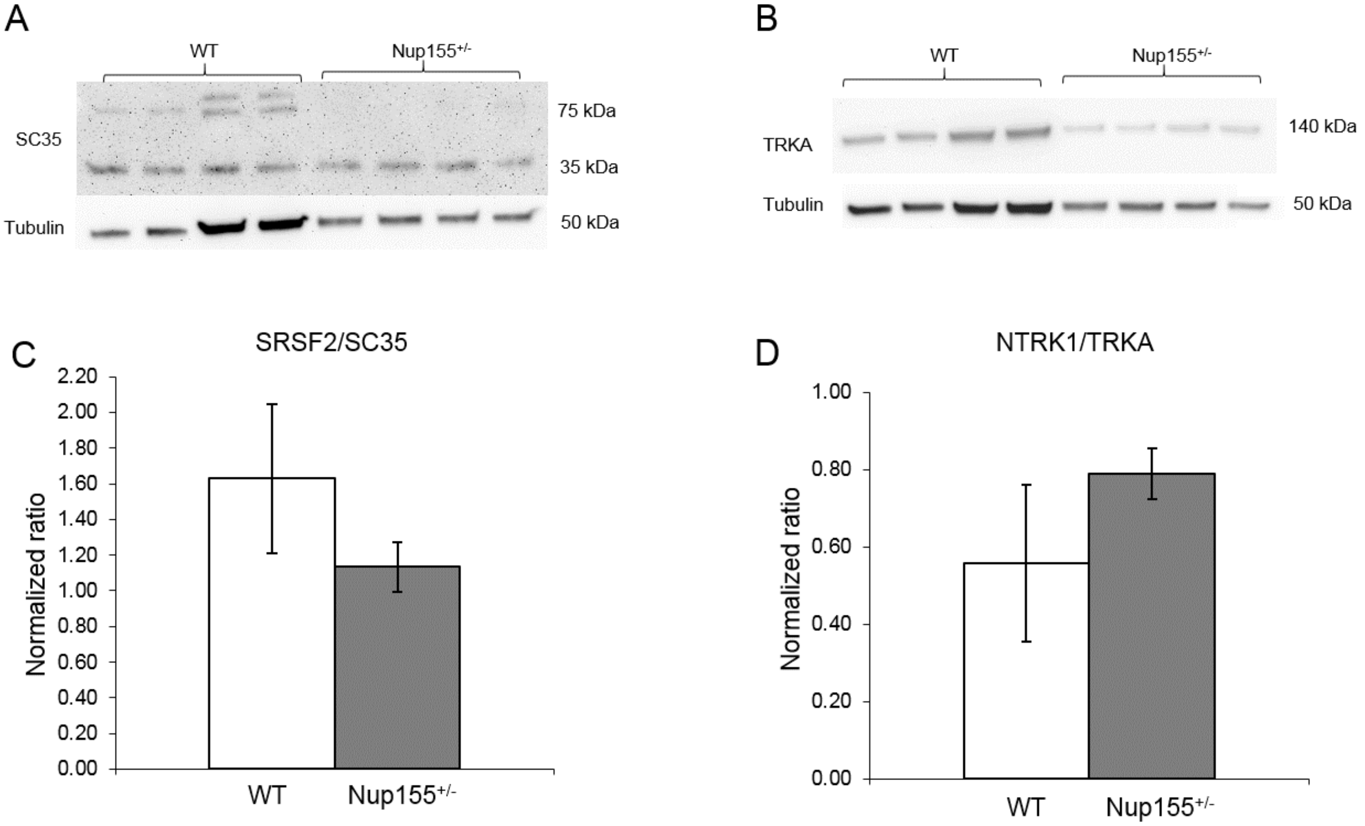

Supplemental Figure. Protein Expression data for SRSF2/SC35 and NTRK1/TRKA. Western blots of (A) SRSF2/SC35 and (B) NTRK1/TRKA from Wild Type (WT, n=4) and Nup155$^{+/-}$ (n=4) ES cell lines. β tubulin was used as loading control for both proteins. Comparison of normalized ratios from densitometry analyses of (C) SRSF2/SC35 and (D) NTRK1/TRKA protein levels in WT and Nup155$^{+/-}$ cell lines ($p > 0.05$).
